# Toward a near real-time magma ascent monitoring by combined fluid inclusion barometry and ongoing seismicity

**DOI:** 10.1126/sciadv.adi4300

**Published:** 2024-02-07

**Authors:** Vittorio Zanon, Luca D’Auria, Federica Schiavi, Klaudia Cyrzan, Matthew J. Pankhurst

**Affiliations:** ^1^ Instituto de Investigação em Vulcanologia e Avaliação de Riscos (IVAR), Universidade dos Açores, Rua Mãe de Deus, 9500-123 Ponta Delgada, Portugal.; ^2^ Instituto Tecnológico y de Energías Renovables (ITER), 38600 Granadilla de Abona, Tenerife, Canary Islands, Spain.; ^3^ Instituto Volcanológico de Canarias (INVOLCAN), 38400 Puerto de la Cruz, Tenerife, Canary Islands, Spain.; ^4^ Laboratoire Magmas et Volcans, CNRS, IRD, OPGC, Université Clermont Auvergne, F-63000 Clermont-Ferrand, France.

## Abstract

Fluid inclusion microthermometry on olivines, clinopyroxenes, and amphiboles was used during a volcanic eruption, in combination with real-time seismic data and rapid petrographic observations, for petrological monitoring purposes. By applying this approach to the study of 18 volcanic samples collected during the eruption of Tajogaite volcano on La Palma Island (Canary Islands) in 2021, changes in the magma system were identified over time and space. Magma batches with distinct petrographic and geochemical characteristics emerged from source zones whose depth progressively increased from 27 to 31 kilometers. The rise of magma of deeper origin is attested by fluid inclusions made of N_2_ and CO, markers of mantle outgassing. Magma accumulation occurred over different durations at depths of 22 to 27 and 4 to 16 kilometers. Time-integrated magma ascent velocities (including ponding times) were estimated at between 0.01 and 0.1 meters per second. This method is cost-effective and quickly identifies changes in the magma system during an eruption, enhancing petrological monitoring procedures.

## Teaser

Combining fluid inclusion and seismic data allows a near real-time petrological monitoring during eruptions.

## INTRODUCTION

Geophysical monitoring of an active magma system informs us of the depths at which the magma is moving (*1*, *2*) but does not provide information about the source of the magmas and/or where they were stored. Moreover, the geophysical data do not reveal the involvement of multiple magmas of different compositions.

Integrating geophysical information with data from erupted products is an effective way to address these challenges (*3*, *4*, *5*), tracking the paths of magmas from the source zones to the surface. To identify the arrival of fresh magma into a magmatic system, frequent analysis of collected volcanic fragments or small lava clots via rapid microchemical analysis is essential. This protocol is particularly useful for frequently active volcanoes and helps monitor fundamental compositional changes in the magmatic system (*6*). In contrast, global-scale investigations aiming to provide detailed temporal and spatial resolution of magmatic processes before and during an eruption require more detailed geochemical characterization (major and trace elements) of whole rock, glassy tephra samples, and silicate melt inclusions, as well as mineral chemistry and isotopic analyses (*5*, *7*, *8*, *9*). These conventional methods are unsuitable for effective monitoring because the generation and interpretation of petrological and geochemical data demands considerable (processing) time. Efforts have recently been made to speed up the analytical procedures and integrate data from geophysics or gas geochemistry. This approach has been used during the 2018 Kilauea eruption (*10*), the 2021 Fagradalsfjall eruption in the Reykjanes peninsula of Iceland (*11*), and the 2021 Tajogaite eruption (*5**,* *12*). However, considerable uncertainties persist in determining magma ponding depths because mineral-melt geothermobarometry, specifically the pyroxene barometer in its original formulation and subsequent improvements (*13*), has an uncertainty range of up to 140 MPa for calibrated data and up to 370 MPa for uncalibrated data (*14*). These pressures correspond to estimated depths of ±4.6 to 6.1 and ±12.2 to 16.1 km, respectively, in a density range from the upper crust (~2350 kg m^−3^) to the mantle (~3100 kg m^−3^). We evaluated a cost-effective, rapid approach to identify the movement of magma beneath the surface during the 2021 Tajogaite eruption at the Cumbre Vieja volcanic system, La Palma, the northernmost island of the Canary Archipelago situated in the Eastern Atlantic Ocean. This approach integrates fluid inclusion (FI) microthermometry in olivines, clinopyroxenes, and amphiboles with daily syn-eruptive seismicity and quick qualitative petrographic observations on samples erupted from 19 September to 13 December 2021.

FI are droplets of fluid trapped inside crystals either during growth or later as a result of recrystallization along healed micro-fractures. A microthermometric study consists in determining the temperatures of phase changes during heating and cooling of FI to obtain the barometric state of crystallization and re-equilibration specific to the crystal under study. This study, when applied to many crystals, allows constraints to be placed on the dynamics and depth of magma ascent and ponding. FI microthermometry is often more accurate than clinopyroxene melt barometry because it can analyze various inclusions in a mineral assemblage from a single sample with a calculated uncertainty range of 30 to 60 MPa (i.e., ±1.2 to 2.7 km), over a density range from the Earth’s crust to the mantle, and requires little sample preparation. FI barometry has been used to model the magmatic system of numerous volcanoes (*15**–**23*) but has never been applied during an eruption. An attempt in this direction was made using Raman microspectroscopy on a smaller set of FI-hosting samples collected after the Tajogaite eruption, showing good correlation with tectonic signals (*24*). Microthermometry and Raman spectroscopy complement each other in characterizing the density and composition of FI. The accuracy of a microthermometric inquiry is notably higher for high-density inclusions (*25*), even if Raman spectrometers are correctly calibrated (*26*). Nonetheless, Raman spectroscopy enables full characterization of the composition of an inclusion.

In this context, data are extracted over time, with the microthermometric data for each sample reflecting integrated information that relies on ponding depths and ascent velocities of the respective magma parcel. In principle, it would be possible to access the preeruptive magma storage depths and the dynamics of the magma ascent path in real time by collecting microthermometric information from multiple snapshots throughout the eruption (*27*).

### Eruption, seismicity, and sample description

**Fig. 1.**
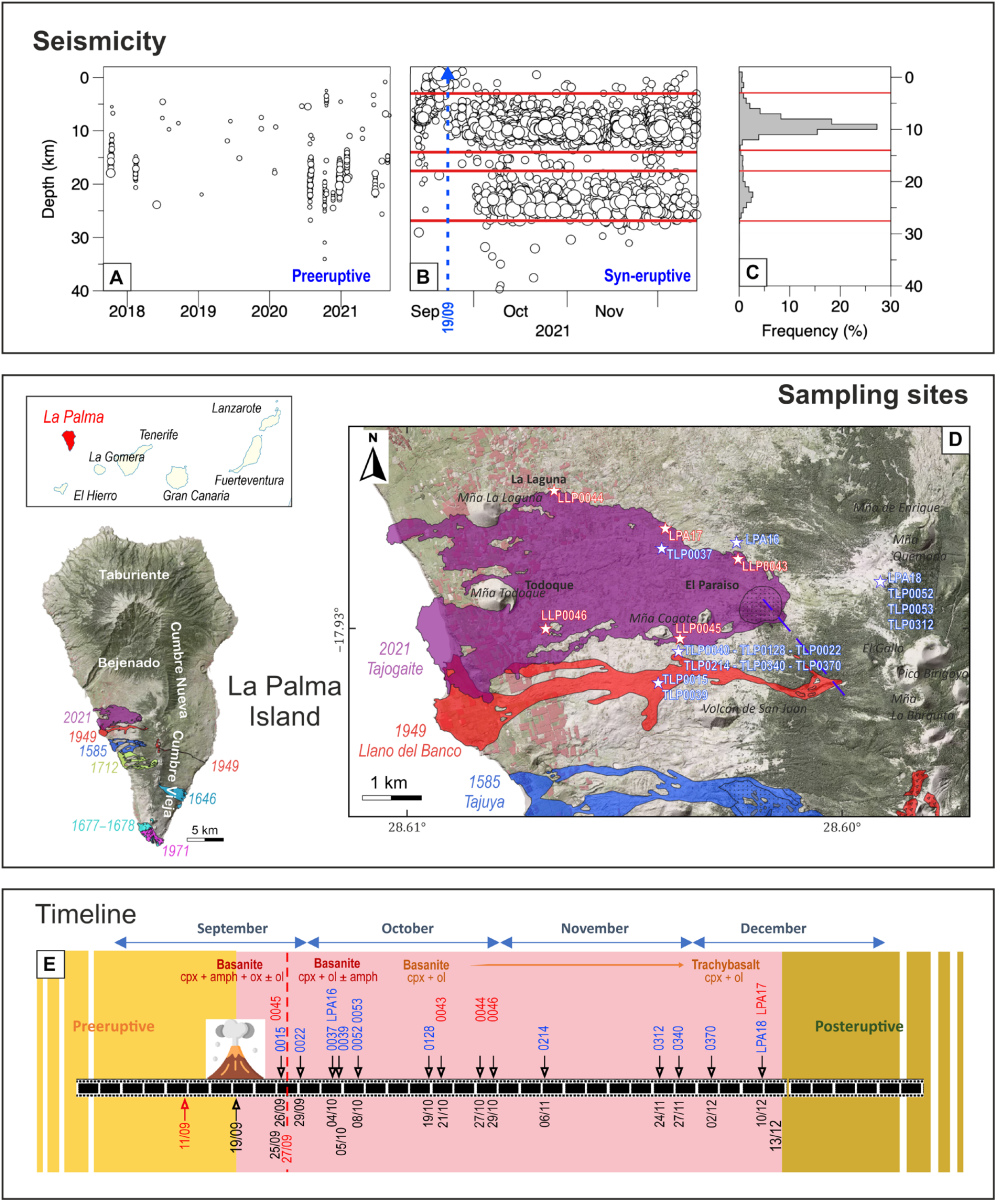
Seismicity, sampling locations, and timeline of volcanic events of the 2021 eruption. Seismic activity before and during the eruption is illustrated in (**A** and **B**), obtained from the open catalog (*28*). The change in hypocenter location is evident just before the eruption commences (B). (**C**) The frequency of seismic events for the two depth regions. The beginning of the eruption is marked by the blue dashed line, while the two depth ranges discussed within the paper, 4 to 14 km and 14 to 30 km, are marked by horizontal red lines. Depths are from the sea level. (**D**) A digital elevation model of La Palma Island highlighting the distribution of the 2021, 1949, and 1585 lavas. The geography of the Canary Islands is presented in the inset. (**E**) The chronogram of the Tajogaite eruption, illustrating the sample collection (indicated by black arrows) and the change in magma composition. The red labels indicate lava samples that were used for whole rock analysis, whereas the blue labels represent tephra samples. On 27 September, the temporary cessation of volcanic activity is marked by a red vertical dashed line.

Eleven distinct seismic swarms of low-magnitude events (*M*_L_ < 2) occurred from October 2017 to early September 2021 (*28*), affecting the entire northern and central sectors of the Cumbre Vieja volcanic system. The hypocentral depths for each seismic swarm generally varied between 15 and 25 km (Fig. 1A). Contextually, the first two seismic swarms were accompanied by geochemical anomalies in the gases emitted at the surface (*29*). All these elements indicated a progressive refilling of the system by multiple magma intrusions at different depths. Starting on 11 September, seismic activity continued with hypocenters initially at a depth of 8 to 9 km and gradually rising at a constant rate up to about 5 km (Fig. 1B). Approximately 1 day before the beginning of the eruption, hypocentral depths migrated rapidly to shallower levels. The latter seismic activity was hydrothermal and subsequently vanished or was obstructed by volcanic tremors (*30*). The eruption began at 14:12 UTC on 19 September and since then syn-eruptive seismicity occurred within two different depth ranges: one at ~18 to 27.5 km (with a mode at 22 km) and another at ~4 to 14 km (with a mode at 9 km) below the volcano (Fig. 1, B and C). Deep seismic activity began to decrease in early December, ~13 days before the cessation of magma emission.

**Fig. 2.**
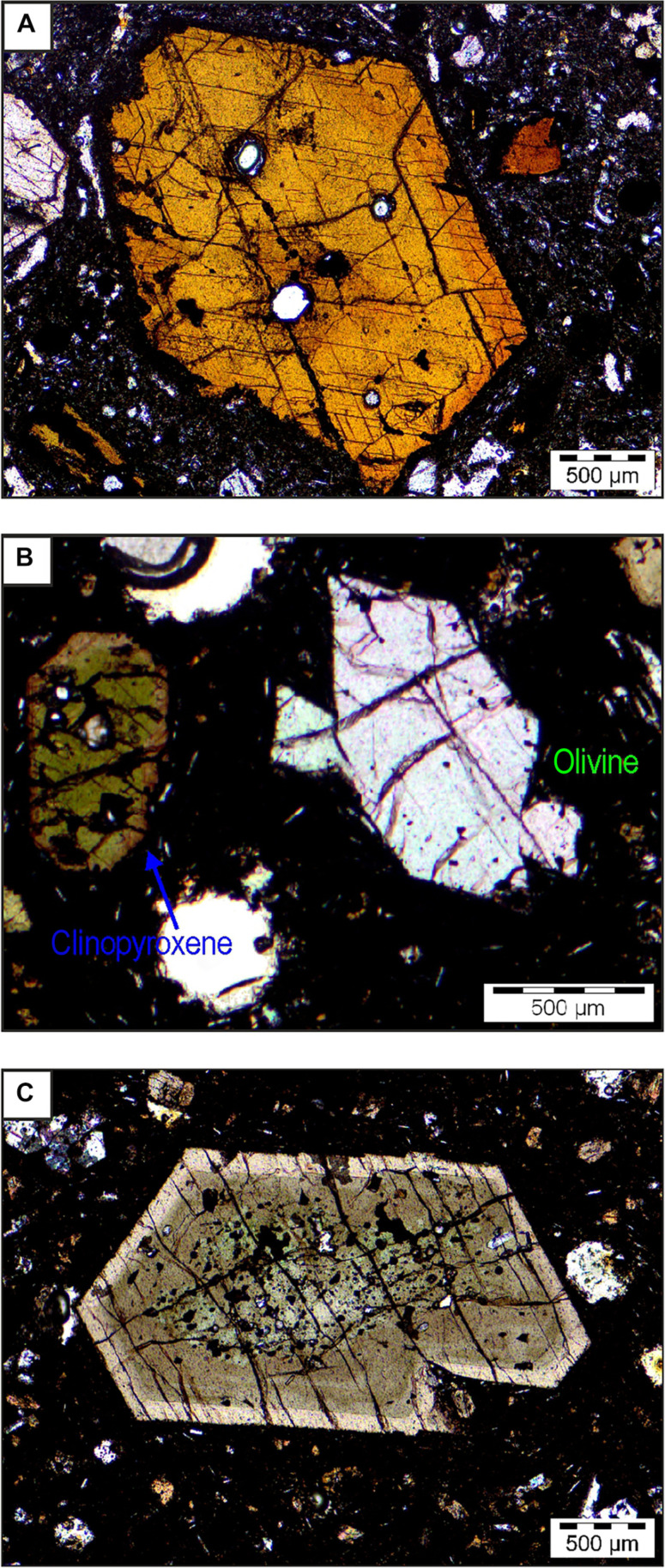
Mineral textures in selected lava samples. (**A**) Zoned amphibole from CAN-LLP-0045 lava from 25 September, showing poorly developed breakdown rims. (**B**) Euhedral/subhedral olivines, also forming an aggregate with small pyroxene and large and partially fragmented clinopyroxene from CAN-LLP-0045. (**C**) Zoned euhedral clinopyroxene with FI from CAN-LLP-0046 lava from 29 October.

Several volcanic vents opened along a north northwest–south southeast trending fissure that stretched for 500 m on the western flank of the Cumbre Vieja ridge at ~950 m above sea level in the area known as “Cabeza de Vaca.” These vents produced a cinder cone that gave rise to a composite lava flow for 3 months (Fig. 1D). Additional information on the volcanological description of the eruption can be found in (*12*, *31*, *32*). A systematic sampling of tephra and lava was conducted during the eruption for petrological monitoring purposes (Fig. 1E). The first magma erupted during the event was a basanite (fig. S1), which contained zoned clinopyroxene, amphibole, titanomagnetite, and rare olivine. This magma displayed a fine-grained groundmass with acicular feldspar. The tephra ejected until 19 October had a tephritic composition (SiO_2_ = 45 to 47 wt % and total alkali = 6.6 to 7.9 wt %) and were composed of glassy fragments. The early samples exhibit euhedral amphibole crystals measuring up to 2.5 mm in diameter with reduced breakdown rims (Fig. 2A), indicating both rapid ascent and equilibrium conditions with the host magma. Aggregates composed of both amphibole and clinopyroxene are sometimes observed. As the eruption progressed, the size of amphibole crystals reduced, and larger reaction rims were formed. After a period of 7 to 10 days, amphibole crystals progressively disappeared from the mineral assemblage and rarely reappeared. Euhedral/subhedral clinopyroxene crystals often several millimeters in size and with zoned rims are common as isolated crystals and in polycrystalline aggregates. These crystals also exhibit notable embayments, irregularly shaped internal cavities, and corroded rims that are indicative of disequilibrium pressure and temperature conditions. Large crystals forming aggregates show oscillatory or patchy zoning, are twinned, and contain oxides. The olivine crystals have a euhedral to subhedral shape and are rarely larger than 1 mm in size (Fig. 2B). Initially, they were scarce, but their abundance increased as the eruption progressed.

On 24 September, there was a decrease in shallow seismic activity, and, on 27 September, magma emission and volcanic tremors stopped for about half a day. Following the resumption of magma emission, more fluid basanites containing zoned clinopyroxene (Fig. 2C) and olivine in a matrix hosting acicular plagioclase were emitted. This clinopyroxene is smaller than earlier crystals, shows oscillatory or patchy zoning, and contains oxides. Skeletal microphenocrysts exhibit normal zoning. The final lava emitted is a basanite at the boundary with trachybasalt (fig. S1A). These lavas are chemically similar to those erupted in 1949 from the Llano del Banco fissure (fig. S1B), aligned with and located only 1.5 km southeast of the Tajogaite cone vents.

## RESULTS

### Fluid inclusions

FI are present in all the examined samples. They were found more frequently in olivine (*N* = 1355), less frequently in clinopyroxene (*N* = 247), and rarely in amphibole (*N* = 59). Early olivines do not contain FI.

**Fig. 3.**
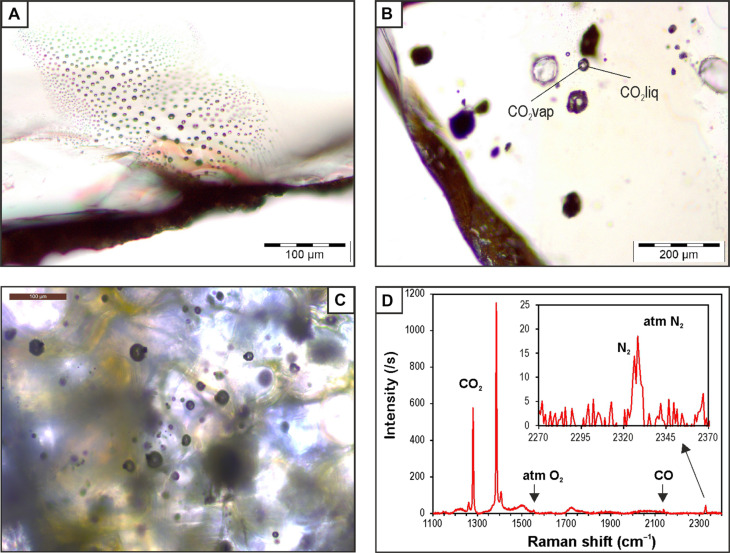
FI textures and Raman data. (**A**) Late-stage FI showing negative crystal shape in olivine from tephra erupted on 5 October. (**B**) Early-stage FI hosted by olivine showing the coexistence of vapor and liquid phases at room temperature. (**C**) Trail of late-stage FI hosted by olivine from 1 December showing the simultaneous trapping of fluid and chromite. (**D**) Raman spectrum of the largest FI shown in (C) containing CO_2_, N_2_, and CO. Raman peaks of O_2_ and N_2_ from the atmosphere are also shown.

Trails of FI crossing the crystals in multiple directions represent the most common texture in all host minerals (Fig. 3A). In olivines, these trails consist of either rounded or negative-crystal shaped inclusions, which are typically 2 to 4 μm in diameter and lack obvious evidence of re-equilibration such as a black rim around the main cavity or tiny radial cracks (*33**–**35*). However, accurate observation is prevented by the small size of the inclusions, so the possible occurrence of a minimal degree of re-equilibration cannot be ruled out. These inclusions were trapped after the host mineral formed during magma ponding events [secondary or late-stage FI, based on textural criteria; (*36*)]. In clinopyroxenes and amphiboles, these trails consist of inclusions with a small variability in size (10 to 15 μm across) in each trail. The inclusions have a rounded to slightly elliptical shape, suggesting some degree of re-equilibration.

In all host minerals, isolated or clustered inclusions of variable size (10 to 40 μm across) are less common (Fig. 3B). These inclusions, trapped during crystallization of the host [primary or early-stage FI, based on textural criteria; (*36*)], often show elongated shapes or a halo of tiny bubbles (<0.5 μm across) surrounding the main cavity, which indicates the occurrence of re-equilibration.

### FI microthermometry

At room temperature, FI are single-phase (either liquid or vapor) or two-phase (liquid + vapor) (Fig. 3B). Amphibole, clinopyroxene, and most olivines contain FI with pure CO_2_ composition and melting temperature of −56.6° ± 0.1°C. Liquid water was not clearly detected either optically or by Raman spectroscopy at room temperature; however, the presence of a few moles cannot be excluded (*37*). Raman spectroscopy revealed the frequent presence of very small crystals of magnesite in FI found in olivines. Its presence suggests that some water was originally present before reacting with the host olivine and fluid CO_2_ to form a carbonate (*38*). It was estimated that the likely original amount of water dissolved in magmas from intraplate settings is around 10 mol % (*39*, *40*). Thermodynamic modeling suggests that, upon cooling and at 0.1 GPa, talc would have formed in FI alongside carbonates from the reaction between forsterite and CO_2_-H_2_O fluids if *X*_H2O_ > 0.1 (*25*). However, talc has not been observed in Raman spectra.

Approximately 5% of the FI hosted in olivines that erupted after 21 October had a melting temperature ranging from −57.4° to −57.0° ± 0.1°C. Some of these inclusions got entrapped alongside chromite (Fig. 3C). The composition of these inclusions, expressed in mole percentage, consists of ~85 to 88% CO_2_, ~10% H_2_O, ~3 to 5% N_2_, and occasionally 0.9% CO (Fig. 3D; details of the molar proportion quantification procedure are given in Materials and Methods). Comparable inclusions were discovered in a spinel-bearing dunite from Lanzarote (*41*).

Final inclusion homogenization occurred to the liquid phase (*Th*_L_) in all minerals and to the vapor phase (*Th*_V_) in a few olivines. Table 1 provides a summary of the homogenization temperatures, densities, and pressures present in the microthermometric database.

**Table 1.** Micrometric database. Data correspond to the day of eruption. Crystals analyzed are olivines (ol), clinopyroxenes (cpx), and amphiboles (amph), and the total number of crystals analyzed is given between parentheses. Homogenization temperatures to the vapor phase are shown in italics. The reported density has been corrected for the possible presence of 10 mol % H_2_O in the inclusion using the method proposed in (*40*).

Sample	Data	Crystals analyzed	No. measures	*Th*_L range_ (°C)	ρ (kg m−3)	*T*_trapping_ (°C)	*P*_max_ (MPa)
				*Th*_V range_ (°C)			
CAN-LLP-0045	25 September	cpx (2)	122	25.6–28.8	664–733	1075	345–424
CAN-TLP-0015	26 September	cpx (1)	56	28.1–30.3	605–683	1075	286–366
CAN-TLP-0022	29 September	ol (2)	12	3.8–16.2	855–945	1150	610–729
LPA-16	4 October	amph (2)	3	23.3–30.9	546–768	1075	240–473
		cpx (1)	2	30.1–30.9	514–615	1075	215–296
		ol (3)	34	13.9–23.7	763–868	1150	493–628
CAN-TLP-0037	4 October	amph (1)	3	26.1–28.7	665–725	1075	349–419
		cpx (1)	21	28.9–30.9	543–661	1075	235–342
		ol (1)	29	6.5–11.4	889–926	1150	656–704
CAN-TLP-0039	5 October	ol (3)	166	1.1–30.9	514–963	1150	227–752
CAN-TLP-0052	8 October	ol (4)	113	1.0–30.9	514–963	1150	227–752
				*30.3–30.4*	*382–388*		*159–167*
CAN-TLP-0053	8 October	ol (1)	1	14.2	865	1150	624
CAN-TLP-0128	19 October	amph (2)	53	23.3–30.8	554–768	1075	246–473
		cpx (1)	46	27.2–30.9	514–703	1075	215–388
		ol (2)	8	12.1–15.9	850–883	1150	603–648
CAN-LLP-0043	21 October	ol (3)	127	−13.0–30.9	496–1043	1150	650–847
				*23.0–30.9*	*230–433*		*91–179*
CAN-LLP-0046	29 October	ol (4)	210	20.5–30.8	536–803	1150	263–540
				*24.9–30.8*	*252–423*		*101–174*
CAN-TLP-0214	6 November	ol (2)	80	−9.7–30.9	496–1023	1150	215–816
				*30.8*	*416*		*171*
CAN-TLP-0312	24 November	ol (5)	143	1.3–0.8	561–961	1150	264–749
CAN-TLP-0340	27 November	ol (9)	103	−8.3–24.3	701–1018	1150	409–818
				*27.7–28.4*	*269–311*		*122–129*
CAN-TLP-0370	2 December	ol (3)	78	−3.9–19.8	811–993	1150	551–789
LPA-17	10 December	ol (6)	153	−13.4–30.8	562–1045	1150	264–865
				*27.8–30.9*	*298–471*		*123–199*
LPA-18	10 December	ol (1)	98	12.5–21.3	794–880	1150	488–644

**Fig. 4.**
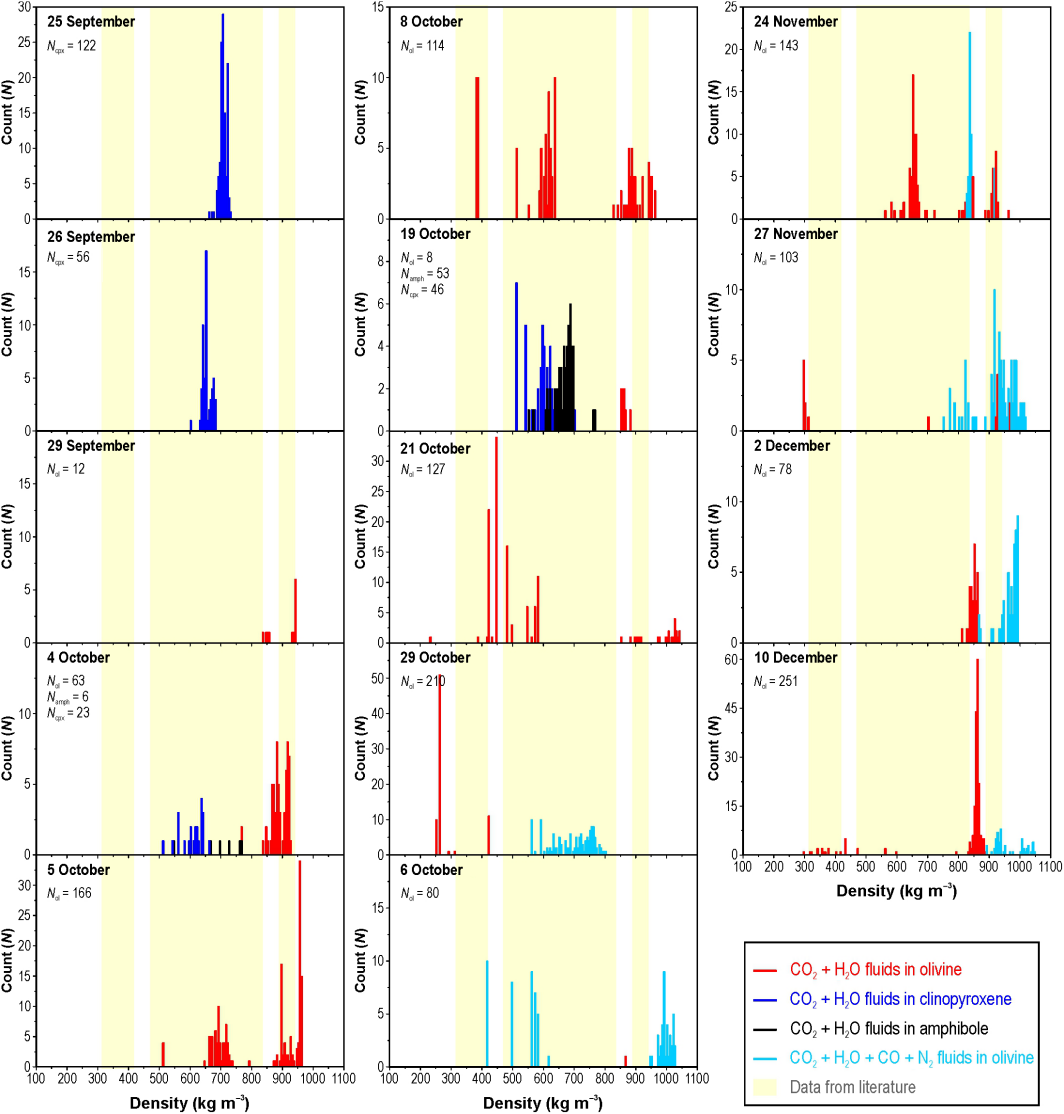
Histograms of FI density data. Data from clinopyroxenes are shown in blue; data from amphiboles are shown in black. Data from CO_2_ + H_2_O–bearing FI in olivines are shown in red; cyan bars are data from CO_2_ + H_2_O + CO + N_2_–bearing FI. Vertical pale yellow stripes represent density ranges from literature data for the whole Cumbre Vieja volcano (*17*).

The density distribution histograms (Fig. 4) and the textural characteristics of FI overall reveal a main trapping event of fluids at depth, followed by a single re-equilibration event during magma ponding at a shallower level. It is assumed that no further re-equilibration occurred during ascent in the conduit. FI populations of fluids trapped in amphiboles and pyroxenes are both early and late-stage and define unimodal or slightly skewed distributions, which can be attributed to inclusion stretching in response to overpressure, developed during rapid and quasi-isothermal ascent (*42*). Although relatively scarce in quantity, the data are coherent with each other. Nearly all FI found in amphiboles (ρ_r_ = 522 to 735 kg m^−3^) are texturally late-stage, except for a few early-stage FI with a density of 636 to 694 kg m^−3^. Similarly, clinopyroxenes from the basanites erupted from mid-October host both early and late-stage FI (ρ_r_ = 514 to 733 kg m^−3^).

The fluids trapped in olivine illustrate polymodal density distributions, with two or three primary modes observed on most sampling days (Fig. 4). Most data range between ~550 and 1045 kg m^−3^. In this density range, small-sized late-stage inclusions with no apparent signs of re-equilibration were found, and skewed distributions are limited to plastic deformation and fluid leakage (*43*). Although there is a possibility of a minimal extent of re-equilibration, these inclusions are still considered good proxies for the original trapping condition. On the other hand, several larger FI exhibiting isolated density peaks show signs of re-equilibration and reduced densities, ranging between 230 and 500 kg m^−3^.

Until 19 October, the FI population with densities ~625 to 730 kg m^−3^ and the olivine-hosted FI population with densities of ~850 to 960 kg m^−3^ have been present, suggesting a well-established magma ascent path in terms of conduits, ponding stages, and ascent rate. The populations of high-density FI gradually increased over time, and an intermittently appearing population of very high-density inclusions (~995 to 1045 kg m^−3^) emerged starting from 21 October. Scattered low-density peaks at around 240, 300, and 370 kg m^−3^ appeared intermittently in olivine-hosted FI from 8 October to 27 November.

The studied samples show no evidence of post-eruptive re-equilibration in slow cooling lavas. For instance, the density histograms of FI in pyroxenes from 25 September lava and 26 September tephra look very similar and exhibit a single mode between 650 and 700 kg m^−3^. The scattered low-density peaks, ranging from 240 to 370 kg m^−3^ and associated with early-stage inclusions, are found in both lavas and tephra samples emitted from October to late November. The comparison between lava and tephra sampled on the last day of the eruption shows a reduced number of late-stage FI re-equilibrated to low pressure in the lava sample. This population characterizes only the FI present in one olivine from the six analyzed lava samples, suggesting the capture of a remnant of a prior magma pulse.

## DISCUSSION

### The architecture of the Cumbre Vieja magmatic system

The barometric information on the magma system was derived from the isochore distribution in P-T space. It was assumed that all FI in clinopyroxenes and amphiboles were either trapped or re-equilibrated at 1075°C, and those in olivines at 1150°C (the reader is referred to Materials and Methods for more details). The magma ascent was considered rapid enough to be treated as isothermal. It was required to create a stratigraphic model beneath the volcano to translate the barometric data into depths. Thus, assumptions were made about the density of the rock bodies and the depth of the transition from crust to mantle (please refer to the Supplementary Materials for more details).

In early to mid-October, two stable modes of FI populations were observed at the ranges of 325 to 420 MPa and 600 to 750 MPa (fig. S2). The high-pressure range corresponds to the same barometric conditions of amphibole and clinopyroxene crystallization and fractionation found in the magmas from the 1949 eruption (*44*). At the end of November, the high-pressure range widened slightly at 590 to 790 MPa, and a population of very high-pressure FI (755 to 865 MPa) emerged intermittently starting from 21 October. The high to very high pressure populations of FI in November and December olivines contain ∼3 to 5 mol % N_2_ and occasionally 0.9 mol % CO (Fig. 3D). In the same samples, other olivines contain FI with only CO_2_ + H_2_O composition and reach a maximum pressure of 740 MPa. Nitrogen is a volatile species that is poorly soluble in an oxidized mantle, such as that beneath the Canary Islands (*45**,* *46*). Under such conditions, early N_2_ degassing from a silicate melt is enhanced at depth (*47**–**49*), leaving silicate melts saturated with CO_2_ and H_2_O. Thus, FI assemblages could track the degassing path of the magma, with the N_2_-bearing population trapped early at depth and simple CO_2_ + H_2_O FI trapped at shallower depths.

The FI populations trapped or partially re-equilibrated at ~300 to 500 MPa are observable until 27 November (fig. S2), and their modes occasionally do not match. The FI found in amphiboles and pyroxenes, along with some olivines erupted until 24 November, return pressures ranging from 200 to 400 MPa. Last, there are some sharp modes, scattered between 75 and 180 MPa, which are associated with early-stage FI and suggest trapping during brief shallow-level magma ponding periods.

Previous microthermometry-based studies on FI at Cumbre Vieja volcano found similar barometric intervals (*17*). This correspondence indicates that the method is reliable and reproducible. Our microthermometric database, which is based on a single eruption, extends to 865 MPa, which is, however, lower than the value of 1140 MPa obtained using clinopyroxene-melt barometry for the 1971 Teneguia eruption (*50*). It is lower than the range of 1.04 to 1.17 GPa found for the old and eroded volcanic edifices of Cumbre Nueva and Taburiente (*51*). This barometric interval recorded by the FI is horizontally distributed under the entire Cumbre Vieja volcano and also extends below the nearby island of El Hierro, highlighting its regional importance (*17*, *52*, *53*).

FI barometry is turned into depths according to the conceptual stratigraphic model presented in fig. S3. These data, in good agreement with the geophysical depths recorded before and during the eruption, indicate that ascending magmas ponded for a longer time at two depth intervals, specifically from −8 to −16 km and from −22 to −27 km, and discontinuously at −4 and −7 km (Fig. 1, B and C, and fig. S4). Both main seismic sources identified sub-vertical volumes that were almost coaxial with a displacement of ~4 km, located near the eruptive fracture (*28**,* *54*) and connected by a dike that dips 19° to 20° northwest. These elements exclude horizontal magma propagation of considerable magnitude, resembling the 2011–2012 El Hierro eruption (*55*). Ascending magmas were temporarily retained and accumulated before their final ascent.

The deepest magma accumulation zone at a depth range of 20.5 to 27.5 km probably consists of mafic to ultramafic cumulate layers (*50**,* *56*) with a ρ = 3115 kg m^−3^, and its lower limit probably marks the transition to the lithospheric mantle (ρ = 3390 kg m^−3^). The lower limit of the intermediate accumulation zone at a depth of 10 to 15 km would mark the transition from rocks with a density of 2655 kg m^−3^ to deeper rocks with a density of 3060 kg m^−3^ (fig. S3). Furthermore, the magmas ponded briefly and intermittently at depths of ~4 km (only in late October) and ~7 km (from October to early November and late December).

### Magma ascent dynamics and velocity

The 2021 Tajogaite eruption ejected multiple batches of mantle-derived magma that ascended through the volcano’s magma system at different rates, as evidenced by the different degrees of re-equilibration (*43**,* *57*) and by the simultaneous presence in the same sample of olivines hosting CO_2_ (+ H_2_O) fluids and olivines hosting CO_2_ + N_2_ (± CO) fluids. The survival of nitrogen-bearing FI depended on the duration of magma ponding at depths near the transition to mantle lithologies at the base of the deeper magma accumulation zone.

**Fig. 5.**
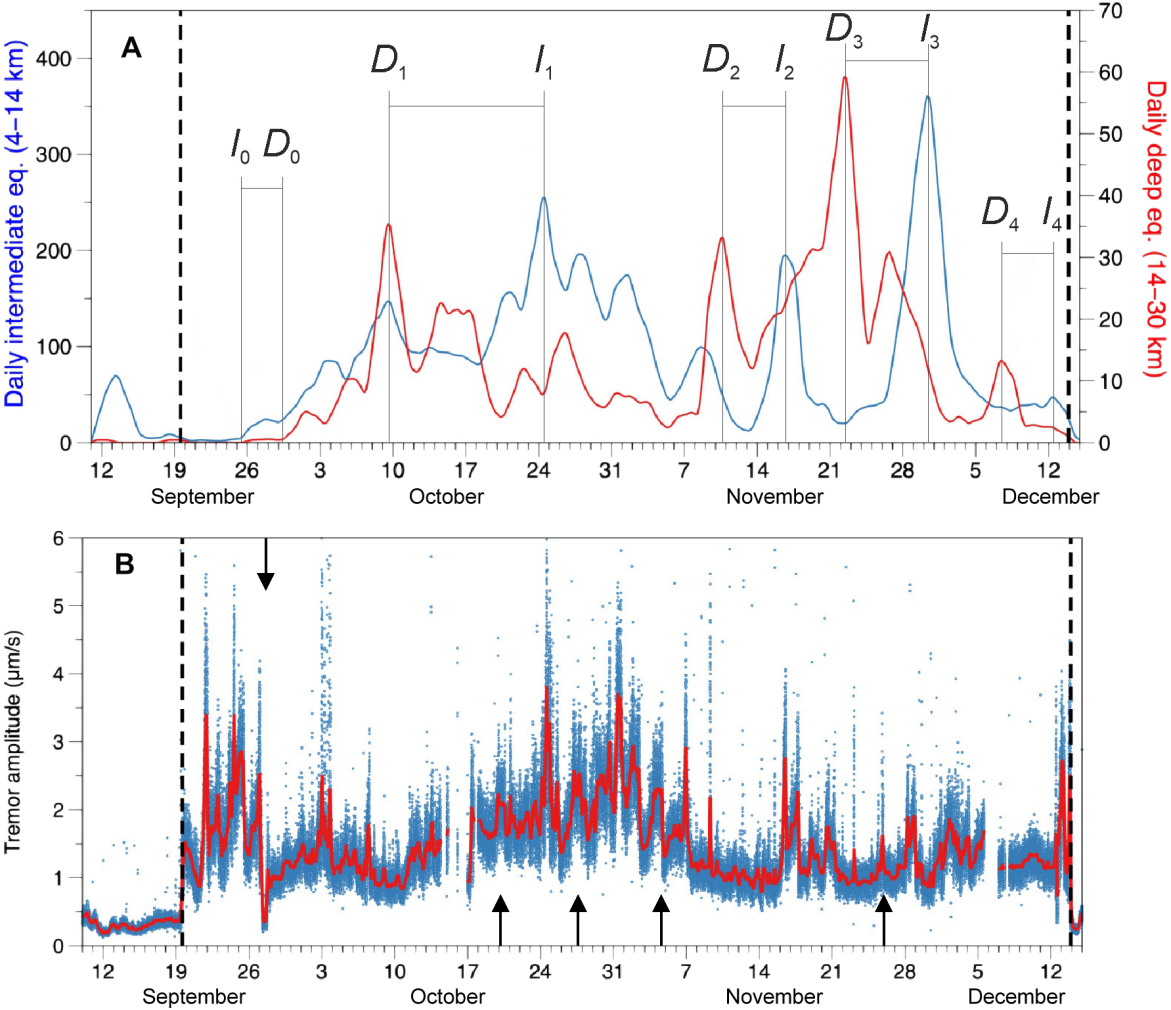
Magma velocity derived from geophysical data. (**A**) The frequency curves of deep (red curve) and shallow (blue curve) earthquakes obtained by a 1-day moving average. On the ordinates, the daily number of earthquakes occurring at different depths is reported. The boundary between the two depth ranges is arbitrary. Velocity is estimated as the time difference between two corresponding frequency peaks as the magma moves from the top of the deep reservoir to the upper reservoir. The codes are explained in Table 2. (**B**) The tremor amplitude pattern (blue dots) and the interpolation of the mean values (red curve). Dashed lines mark the beginning and end of magma emission. Velocity is estimated as the time difference between high-amplitude tremor events (black arrows) and the day of sampling, when FI indicated a short and very shallow ponding stage (between 4.3 and 7 km). Relevant data are presented in Table 2.

Given the architecture of the magma system, the preeruptive seismicity was related to magma refilling of existing structures at depth. Regarding syn-eruptive seismicity, two well-separated clusters were observed (*28*) and justified with the readjustment of the crust and upper mantle due to the emptying of these two magmatic reservoirs. Figure 5A clearly shows the temporal relationship between the deep and intermediate seismicity, confirming some kind of hydraulic connection between these two reservoirs. This hypothesis is also consistent with the results of (*5*). This process may have induced a downward piston effect (*28*) that temporarily halted magma withdrawal during periods of compression and enhanced brief magma ponding. This piston effect could be responsible for the deepening of the magma source over time, as revealed by FI barometry. A similar process was already observed in the recent Fagradalsfjall eruption (*11*) and was accompanied by an increase in the flow rate.

The similarity of the earthquake frequency curves generated in the two storage areas (Fig. 5A), along with the temporal shift of similar frequency peaks, can provide estimates of the total number of magma pulses that occurred (method 1). These observations may additionally provide an estimate of the time-integrated magma ascent velocity between the two main ponding zones, which includes the residence time, as indicated in Table 2. To avoid any possible misinterpretation, we only considered peaks whose amplitude clearly stands out from the surrounding values and that show a similar shape in the two curves. Using this conservative approach is essential to ensure the accuracy of our qualitative estimates. Moreover, the correlation between intermittent magma ponding at depths of ~4 and ~7 to 8 km with peaks of high tremor amplitude in Fig. 5B can facilitate the calculation of the final magma velocity (method 2). In this diagram, the very long period component of tremor, which seems to be related to the volume of gas involved, has been used. We hypothesize that the shallow ponding mentioned in this context could be related to the accumulation of gas, which ultimately fueled the energetic episodes of lava fountains.

**Table 2.** Time-integrated magma ascent velocity between seismic zones. Seismic references are shown in Fig. 5. ∆*X* of 13.5 km is the distance between the tops of the two magma accumulation zones, whereas ∆*X* is the distance between the shallowest magma ponding zone and the sea level in the remaining part of the table.

Seismic reference	Date	Estimated time	Possible interpretation	∆*X* (m)	*v*_av_ (m s−1)
*I*_0_	25 September	3 days, 20 hours	The decompression caused by magma withdrawal from 4 to 14 km depth (*I*_0_) causes the mobilization of deeper magma batch from the deeper reservoir (*D*_0_) to compensate the pressure gradient.	13,500	0.04
*D*_0_	29 September	(331,200 s)			

*D*_1_	9 October	14 days, 22 hours	At the end of deep magma withdrawal (*D*_1_), possible gravitative adjustments caused a negative pressure gradient propagating upward, affecting the conduit system at 4- to 14-km depth (*I*_1_).	13,500	0.01
*I*_1_	24 October	(1,288,800 s)			

*D*_2_	10 November	8 days	At the end of deep magma extraction (*D*_2_), possible gravitative adjustments caused a negative pressure gradient propagating upward, affecting the conduit system at 4- to 14-km depth (*I*_2_).	13,500	0.02
*I*_2_	16 November	(691,200 s)			

*D*_3_	22 November	6 days, 1 hour	At the end of deep magma extraction (*D*_3_), possible gravitative adjustments caused a negative pressure gradient propagating upward, affecting the conduit system at 4- to 14-km depth (*I*_3_).	13,500	0.03
*I*_3_	30 November	(522,000 s)			

*D*_4_	7 December	4 days, 23 hours	Following the last event of magma withdrawal (*D*_4_), possible gravitative adjustments caused a negative pressure gradient propagating upward, affecting the conduit system at 4- to 14-km depth (*I*_4_).	13,500	0.03
*I*_4_	12 December	(428,400 s)			

Tremor amplitude	27 September	2 days, 8 hours	Temporary end of tremor	15,000	0.07
*I*_0_	25 September	(201,600 s)	Beginning of decompression of the intermediate reservoir		

Tremor amplitude	20 October	12 hours	Peak of tremor amplitude	4,300	0.1
Sample collection day*	21 October	(43,200 s)	FI recording shallow ponding stage		

Tremor amplitude	27 October	24 hours	Peak of tremor amplitude	4,300	0.05
Sample collection day*	29 October	(86,400 s)	FI recording shallow ponding stage		

Tremor amplitude	4 November	1 days, 10 hours	Peak of tremor amplitude	7,000	0.06
Sample collection day*	6 November	(122,400 s)	FI recording shallow ponding stage		

Tremor amplitude	25 November	16 hours	Peak of tremor amplitude	5,000	0.09
Sample collection day*	27 November	(57,600 s)	FI recording shallow ponding stage		

*A delay of 12 hours was applied between tephra emission and sample collection.

The velocities obtained by these two independent methods are summarized in Table 2 and range from 0.01 to 0.04 m s^−1^ from the deep to the intermediate magma accumulation zone and from 0.05 to 0.1 m s^−1^ from the shallower, intermittently active ponding zone at 4- to 7-km depth. The velocities listed above are minimum values because it is difficult to determine the precise timing of tephra emission and the exact depth of seismicity corresponding to each peak. The variability of these estimates could be partially explained by the presence of volumetrically different pulses of magma ascending through a magma system, which changes with time, by the dynamics of magma extraction that experiences varying degrees of pressurization and decompression, and by different ponding times. These estimates are consistent with the absence of dense ultramafic xenoliths in the erupted products, indicating that an ascent rate greater than 0.2 m s^−1^ is required for a bubble-free melt, as suggested by (*58*). The estimated time-integrated velocities mentioned above are an order of magnitude lower compared to the ascent velocity calculated for xenolith-bearing basanites during the 1949 eruption (*59*).

### Near real-time magma ascent monitoring

**Fig. 6.**
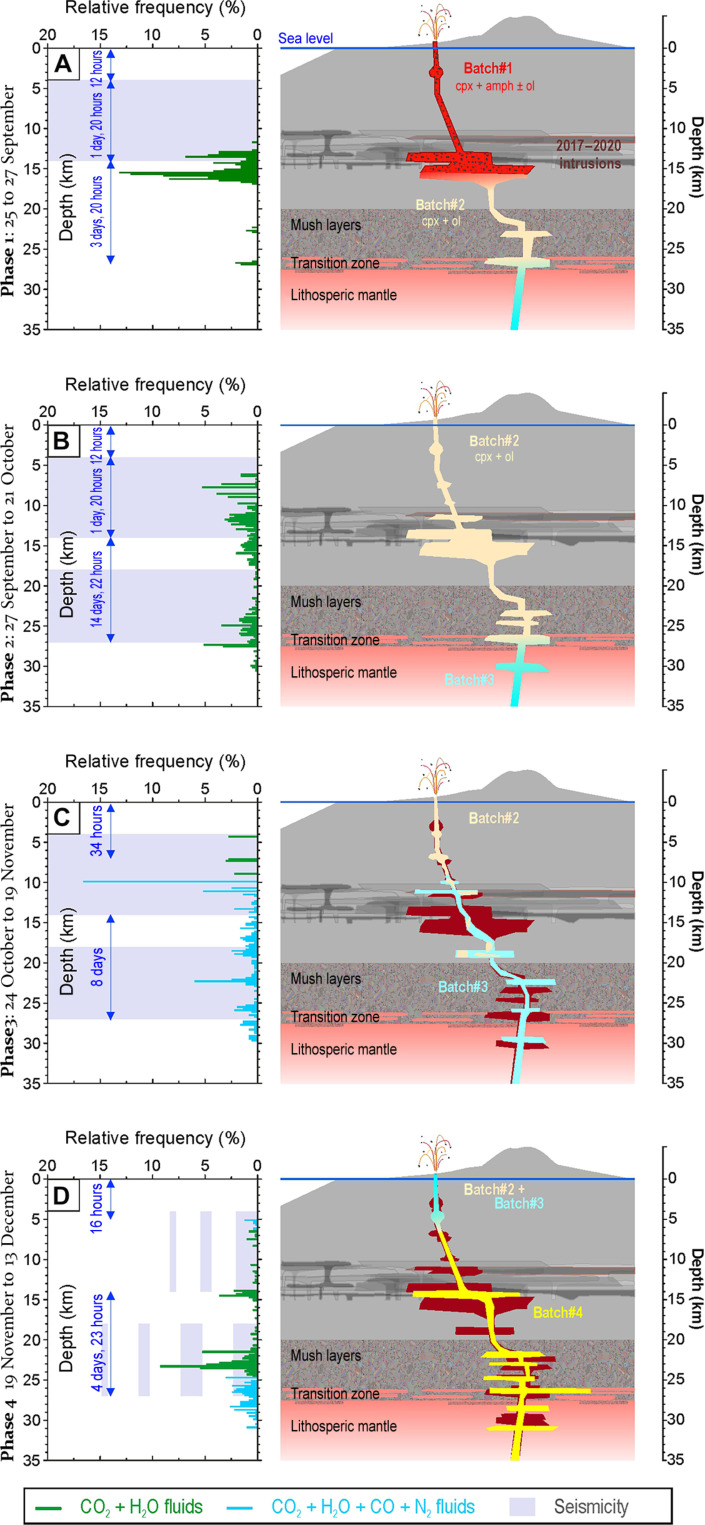
Cartoon resuming the main periods of magma ascent monitoring under the Tajogaite volcano, in 2021. (**A** to **D**) Histograms of the frequency of depths have been normalized to 100%. Green bars represent CO_2_ + H_2_O FI (hosted in clinopyroxene, amphibole, and olivine); cyan bars represent olivine-hosted CO + N_2_ (+ CO_2_ + H_2_O)–bearing FI. Light-violet horizontal stripes represent seismicity (*28*). These stripes, when dashed, indicate decreasing seismicity. The estimated ascent time is shown in blue. The cartoons on the right show the conceptual model with the different ascending magma batches (in different colors) and their ponding stages.

The histograms of magma ponding depths (fig. S4) were deconstructed as a function of the various ascent rates between the two principal zones of magma accumulation to describe how the magma system’s dynamics evolved during the eruption as multiple pulses of magma ascended (Fig. 6).

Phase 1: From 19 to 27 September (Fig. 6E). Preeruptive activity included a series of low-magnitude seismic swarms lasting a few days and ranging in depth from 15 to 25 km (Fig. 1A), highlighting multiple magma intrusions. During its ascent, the 11 September intrusion displaced a cool and partially evolved basanite residing between ~13- and 16.5-km depth. The latter magma, the first to be erupted (batch#1), was either a remnant of the 1949 Llano del Banco eruption or, more likely, one of the earliest intrusions in 2017, whose amphibole and clinopyroxene had sufficient time to fully re-equilibrate their FI populations. The calculated ascent rate of 0.04 m s^−1^ (Table 2) allows to define that all olivines sampled on 29 September belonged to the 11 September magma (batch#2) and showed a nonstop ascent from a depth of 27 km, as confirmed by the absence of any re-equilibration at shallower depths. Seismicity during this period occurred within the shallower 4 km and in the intermediate seismic zone (Figs. 1A and 5A) and was associated with the emptying of the intermediate magma accumulation zone at a rate of 0.04 m s^−1^. The cessation of seismicity at 4- to 14-km depth on 24 September, followed by the temporary cessation of magma emission and seismic tremor, suggested that the last 14 km of the magma system had been emptied.

Phase 2: From 27 September to 24 October (Fig. 6B). In the early afternoon of 27 September, the eruption resumed with the emission of a hotter basanite (batch#2) whose mineralogy was characterized by a progressive reduction in pyroxene and amphibole crystals and an increase in olivines. In early October, the deeper seismic source was activated, indicating magma ponding at depths of 25 to 27 km. FI barometry still confirms the source of pyroxenes and amphiboles at a depth range of ~13 to 16.5 km (Fig. 6), while olivine rose from a depth of ~22 to 27 km (Fig. 4), but with partial re-equilibration in the intermediate storage zone. The duration of these re-equilibration events was responsible for the estimated slow velocity between the two magma accumulation zones (0.01 m s^−1^).

Phase 3: From 24 October to 19 November (Fig. 6C). During this period, the magma system of Tajogaite volcano was partially occupied by a new pulse of basanite (batch#3), characterized by olivines hosting CO_2_ + H_2_O + CO + N_2_–bearing FI and ascending with an integrated velocity of 0.02 m s^−1^. The presence of olivines hosting CO-N_2_–free FI at the same time suggests that remaining amounts of the prior magma (batch #2) with slow mobility were still present in the conduit (see Fig. 5A). Noteworthy, the contemporaneous presence of these two kinds of magma inside the volcano is confirmed by the decoupling of Sr isotope compositions in matrix melt (*5*) and in the split in Os isotope compositions in whole rock (*12*). The distinctive depths of the two magma pondings and the elevated seismic activity in this period suggest the possible activation of a secondary pathway, which would allow the two magmas to ascend independently.

Phase 4: From 19 November to 13 December (Fig. 6D). From mid-November, seismicity began to decrease at all depths (Fig. 5A). The amplitude of tremor was sustained until early December. FI barometry shows active magma ponding in the intermediate magma accumulation zone only in late November. N_2_-bearing FI and N_2_-free FI in olivines continued to be erupted, possibly through the dual ascent path. The time-integrated ascent velocity during this phase was calculated to be 0.03 m s^−1^, in agreement with the absence of magma ponding in the intermediate accumulation zone. The magma erupted toward the end of the eruption is an alkali basalt (more silicic, but with the same alkali content as the basanites), which forms the batch#4 and ascended from a depth of 31 km. Starting from 1 December, the deep seismic activity came to an end and the intermediate seismic activity continued to decrease. This marked the end of magma supply from the mantle, along with the progressive emptying of the entire magma system of the volcano.

In conclusion, FI microthermometry combined with basic petrographic observations in tephra and lava samples, along with seismicity recorded during the 2021 eruption of Cumbre Vieja, has enabled near real-time monitoring of the ascent of various magma pulses. The consistency of our database, based on 18 time-constrained samples, has highlighted the rise of different magma pulses over 3 months. The geobarometric information on magma ponding from FI analysis correlated precisely with the location of magma extraction from seismogenic sources and indicated the progressive deepening of the magma source (from 27- to 31-km depth) during the eruption.

This combined approach facilitated the reconstruction of the syn-eruptive changes in the magma system of the volcano and provided estimates of the time-integrated magma velocities (from 0.01 to 0.1 m s^−1^), which include pauses in the magma accumulation zones. This information is crucial to comprehend the overall dynamics of magma ascent and to interpret geophysical signals accurately. The high resolution of the data provided in this study is related to the investigation of time-constrained samples and the high number of inclusions analyzed per sample, regardless of the mineral host.

Similar conclusions have been provided in other works, performing Raman spectroscopy on FI (*24*) and a chemical study on clinopyroxene combined with clinopyroxene-melt barometry (*5*), indicating that the study of time-constrained samples is the way for a more efficient petrological monitoring. However, the ability to trace magma ponding events in a short time, due to the reduced time required for sample preparation and analysis, which can be performed in near real-time (*27*), makes FI microthermometry a rapid, concise, and informative method. Its combination with geophysical monitoring makes its use highly recommended for cost-effective petrological monitoring of ongoing magmatic processes during an eruption and represents an advance for the improvement of monitoring strategies, especially in institutions where large analytical platforms are not feasible.

## MATERIALS AND METHODS

### Seismicity

Both the Instituto Geográfico Nacional (IGN) and the Instituto Volcanológico de Canarias (INVOLCAN) recorded seismic monitoring data during the eruption. This work uses the catalog produced by INVOLCAN [publicly available dataset; (*28*)]. The catalog contains hypocenters that have been relocated in real-time during the eruption in a three-dimensional tomographic model, resulting from the joint analysis of seismic phases from both IGN and INVOLCAN. This leads to more reliable depths of the earthquakes.

### Sample description

A total of 13 tephra and five lava samples were collected during the joint routine monitoring of the Tajogaite eruption by the INVOLCAN and the Instituto de Investigação em Vulcanologia e Avaliação de Riscos. A brief description and coordinates are given in table S1.

### Whole-rock and mineral chemistry

Major element compositions of five lava samples differing in petrographic characteristics were analyzed at Actlabs (Activation Laboratories, Canada) using a PerkinElmer 9000 inductively coupled plasma–mass spectrometer and an Agilent 735 inductively coupled plasma–atomic emission spectrometer. Alkaline dissolution with lithium metaborate/tetraborate followed by nitric acid was used on 1 g of rock powder before melting in an induction furnace. The melt was poured into a 5% nitric acid solution containing cadmium as an internal standard and stirred until complete dissolution. The resulting analytical accuracy is better than 6% for all major elements. Seven international rock standards were used to calibrate the two methods. Compositional data for the major elements are given in table S2.

The major element compositions of olivines and clinopyroxenes were measured using a Cameca SXFive electron microprobe (Camparis, Paris, France) at 15 keV and a 20-nA focused beam. Counting times were 30 s on the peak and 10 s on each background. The San Carlos olivine and the Puy de Dôme clinopyroxene, used as standards, were calibrated to within 2% at 2σ. Raw data were corrected using a Phi-Rho-Z quantitative analysis program. The typical detection limit for each element is 0.01%. Relative errors are better than 6% for NiO, alkali, and MnO and better than 2% for all other major elements.

Eruptive temperatures of early basanite magmas were calculated from the MgO content of glass (*60*). Glassy fragments of lapilli ejected in October were analyzed using a Cameca SXFive electron microprobe (Laboratoire Magmas et Volcans, University of Clermont-Auvergne, France) at 15 keV and a 10-nA beam with a defocused spot size of 10 μm. ALV-98I and CH98-DR11 international standard glasses were used to check the errors and reproducibility. Relative errors are better than 3% for most major elements, 19% for MnO, and 25% for K_2_O. Microprobe averages, number of measurements, and SD are given in table S3.

### Fluid inclusions

FI were identified in 18 samples of lava and tephra. The lava samples were initially crushed roughly using a jaw crusher. The crushed lavas and tephra were then sieved to isolate different crystal size populations. For every sample, around 100 olivines (diameters ranging from 0.35 to 0.65 cm), 50 clinopyroxenes, and amphiboles (up to 1.0 cm long) were separated. These separated crystals were then thinned to a thickness of 60 to 80 μm, double polished, and examined under a light microscope to search for FI. The inclusions were identified in all of the samples. Microthermometry was conducted on a Linkam MDSG600 heating-cooling stage, which was calibrated with synthetic FI standards consisting of pure CO_2_ and H_2_O. Reproducible melting and homogenization temperatures to within ±0.1°C were obtained at heating rates of 0.2° to 0.5°C/min.

The density values of the CO_2_ fluid were computed according to equations 3.14 and 3.15 of (*61*). Isochores were obtained for a pure CO_2_ fluid using the equation of state for carbon dioxide of (*62*), which is valid up to at least 2000 K and 10 GPa. High density values for pure CO_2_ fluids in olivine were corrected for a probable pristine presence of ~10% water [H_2_O:CO_2_ = 1:9; (*40*)] and then isochores for these H_2_O-CO_2_ fluids were calculated (*63*). When recording the presence of N_2_ and CO, isochores were computed only for inclusions having negative homogenization temperature using *VX* plots (*64*). On the basis of this study’s findings, at positive homogenization temperatures, the impact of increasing bulk density in FI up to 5% N_2_ in the mixture is trivial.

The eruption temperatures for early magmas have been calculated using chemical geothermometry (*60*) modified by (*13*), which correlates the temperature of olivine crystallization with the MgO concentration of the magma. The temperatures ranged from 1073° to 1084°C (table S3). The temperature of 1075°C was applied to calculate the isochores of FI that were trapped in clinopyroxenes and amphiboles. For those that were trapped in olivines, a temperature of 1150°C was used in compliance with direct field measurements and experimental petrology data for magmas erupted in December (*12*, *32*, *65*). Uncertainty on the eruptive temperature calculation has a little effect on the final pressure calculation: Considering an FI with ρ = 1000 kg m^−3^, an uncertainty of ±20°C in the estimation of the eruptive temperature (e.g., between 1130° and 1150°C) generates a maximum error of ±10 MPa.

Table 1 presents microthermometric data, while fig. S2 shows the histograms of trapping/re-equilibration pressures. Using the stratigraphic scheme described in the Supplementary Materials and shown in fig. S3, pressures were converted into depths. In this model, we defined major stratigraphic changes through both the interpretation of the seismic velocity model in (*28*, *54*) and the FI ponding stage scheme (this work). After measurements using an electronic densimeter, we assigned rock density values to shallow lavas (ρ = 2350 kg m^−3^), dense gabbroic xenoliths (ρ = 3655 kg m^−3^), and mantle lithologies (ρ = 3115 to 3390 kg m^−3^), while we assumed the density values for other rock bodies.

### Raman microspectroscopy

Raman microspectroscopic analysis was performed on two olivine crystals from a lava sample collected at the end of the eruption (10 December) that contain FI with melting temperatures ranging from -56.8° to -57.3°C, indicating the presence of volatile species other than CO_2_ and H_2_O. FI were analyzed using a Renishaw inVia confocal Raman microspectrometer, equipped with a 532.1 ± 0.3–nm diode-pulsed solid-state laser (~180-mW output power), a 1040 × 256 pixel charge-coupled device detector, a Rayleigh rejection edge filter, and a Leica DM 2500 M optical microscope with a motorized XYZ stage at the Laboratoire Magmas et Volcans (France). The spectra were acquired in backscattered geometry, in both standard and high confocality modes (slit aperture of 65 and 20 μm), with a 50× microscope objective and a grating of 2400 lines/mm. Each acquisition consisted of two accumulations of 20 s, and the laser power was set to 10 or 50% (i.e., ~12 and 60 mW). Spectra were recorded in extended mode from 60 to 4500 cm^−1^ using WiRE 4.4 software. Daily calibration of the spectrometer was performed using a silicon standard (520.5 cm^−1^ peak) and several neon lines. Analyses were carried out at a constant temperature of ~20.5°C.

Following the method described in (*66*), the molar proportions of the different components present in the FI (CO_2_, CO, and N_2_) were calculated from Raman peaks areas (normalized for laser power and exciting time); instrumental efficiencies of 1, 1, and 0.5 for N_2_, CO, and CO_2_, respectively; and wavelength-dependent Raman scattering efficiencies. The wavelength-dependent Raman scattering efficiencies for specific Raman shifts were calculated with respect to the scattering efficiency of N_2_, based on equation 1 of (*66*). CO_2_ concentrations were calculated using the sum of the areas of the two Fermi diad peaks and the sum of their scattering efficiencies.

## Supplementary Materials

### This PDF file includes:

Supplementary Text Figs. S1 to S4 Tables S1 to S4 References

## References

[1] A. García, A. Fernández-Ros, M. Berrocoso, J. M. Marrero, G. Prates, S. De la Cruz-Reyna, R. Ortiz, Magma displacements under insular volcanic fields, applications to eruption forecasting: El Hierro, Canary Islands, 2011–2013. Geophys. J. Int. 197, 322–334 (2014).

[2] Z. Duputel, O. Lengliné, V. Ferrazzini, Constraining spatiotemporal characteristics of magma migration at Piton de la Fournaise volcano from pre-eruptive seismicity. Geophys. Res. Lett. 46, 119–127 (2019). 31423032 10.1029/2018GL080895 PMC6686716

[3] M. J. Pankhurst, D. J. Morgan, T. Thordarson, S. C. Loughlin, Magmatic crystal records in time, space, and process, causatively linked with volcanic unrest. Earth Planet. Sci. Lett. 493, 231–241 (2018).

[4] D. J. Rasmussen, T. A. Plank, D. C. Roman, J. A. Power, R. J. Bodnar, E. H. Hauri, When does eruption run-up begin? Multidisciplinary insight from the 1999 eruption of Shishaldin volcano. Earth Planet. Sci. Lett. 486, 1–14 (2018).

[5] T. Ubide, Á. Márquez, E. Ancochea, M. J. Huertas, R. Herrera, J. J. Coello-Bravo, D. Sanz-Mangas, J. Mulder, A. MacDonald, I. Galindo, Discrete magma injections drive the 2021 La Palma eruption. Sci. Adv. 9, eadg4813 (2023). 37406116 10.1126/sciadv.adg4813 PMC10321733

[6] R. Corsaro, L. Miraglia, V. Zanon, Petrologic monitoring of glasses in the pyroclastites erupted in February 2004 by the Stromboli Volcano, Aeolian Islands, Southern Italy. J. Volcanol. Geotherm. Res. 139, 339–343 (2005).

[7] S. A. Halldórsson, E. Bali, M. E. Hartley, D. A. Neave, D. W. Peate, G. H. Guðfinnsson, I. Bindeman, M. J. Whitehouse, M. S. Riishuus, G. B. M. Pedersen, S. Jakobsson, R. Askew, C. R. Gallagher, E. R. Guðmundsdóttir, J. Gudnason, W. M. Moreland, B. W. Óskarsson, P. Nikkola, H. I. Reynolds, J. Schmith, T. Thordarson, Petrology and geochemistry of the 2014–2015 Holuhraun eruption, central Iceland: Compositional and mineralogical characteristics, temporal variability and magma storage. Contrib. Mineral. Petrol. 173, 1–25 (2018).31983758

[8] P. Landi, R. A. Corsaro, L. Francalanci, L. Civetta, L. Miraglia, M. Pompilio, R. Tesoro, Magma dynamics during the 2007 Stromboli eruption (Aeolian Islands, Italy): Mineralogical, geochemical and isotopic data. J. Volcanol. Geotherm. Res. 182, 255–268 (2009).

[9] N. Spilliaert, P. Allard, N. Métrich, A. V. Sobolev, Melt inclusion record of the conditions of ascent, degassing, and extrusion of volatile-rich alkali basalt during the powerful 2002 flank eruption of Mount Etna (Italy). J. Geophys. Res. Solid 111, B04203 (2006).

[10] C. Gansecki, R. L. Lee, T. Shea, S. P. Lundblad, K. Hon, C. Parcheta, The tangled tale of Kīlauea’s 2018 eruption as told by geochemical monitoring. Science 366, eaaz0147 (2019). 31806782 10.1126/science.aaz0147

[11] S. A. Halldórsson, E. W. Marshall, A. Caracciolo, S. Matthews, E. Bali, M. B. Rasmussen, E. Ranta, J. Gunnarsson-Robin, G. H. Guðfinnsson, O. Sigmarsson, J. Maclennan, M. G. Jackson, M. J. Whitehouse, H. Jeon, Q. H. A. van der Meer, G. K. Mibei, M. H. Kalliokoski, M. M. Repczynska, R. H. Rúnarsdóttir, G. Sigurðsson, M. A. Pfeffer, S. W. Scott, R. Kjartansdóttir, B. I. Kleine, C. Oppenheimer, A. Aiuppa, E. Ilyinskaya, M. Bitetto, G. Giudice, A. Stefánsson, Rapid shifting of a deep magmatic source at Fagradalsfjall volcano, Iceland. Nature 609, 529–534 (2022). 36104557 10.1038/s41586-022-04981-x PMC9477742

[12] J. M. Day, V. R. Troll, M. Aulinas, F. M. Deegan, H. Geiger, J. C. Carracedo, G. Gisbert Pinto, F. J. Perez-Torrado, Mantle source characteristics and magmatic processes during the 2021 La Palma eruption. Earth Planet. Sci. Lett. 597, 117793 (2022).

[13] K. D. Putirka, “Thermometers and barometers for volcanic systems” in *Minerals, Inclusions and Volcanic Processes*, vol. 69 of Reviews in Mineralogy Geochemistry, K. D. Putirka, F. Tepley, Eds. (Mineralogical Society of America, 2008), pp. 61–120.

[14] P. E. Wieser, A. J. F. Kent, C. B. Till, J. Donovan, D. A. Neave, D. L. Blatter, M. J. Krawczynski, Barometers behaving badly: Assessing the influence of analytical and experimental uncertainty on clinopyroxene thermobarometry calculations at crustal conditions. J. Petrol. 64, egac126 (2022).

[15] T. H. Hansteen, A. Klügel, H.-U. Schmincke, Multi-stage magma ascent beneath the Canary Islands: Evidence from fluid inclusions. Contrib. Mineral. Petrol. 132, 48–64 (1998).

[16] E. Hildner, A. Klügel, T. H. Hansteen, Barometry of lavas from the 1951 eruption of Fogo, Cape Verde Islands: Implications for historic and prehistoric magma plumbing systems. J. Volcanol. Geotherm. Res. 217–218, 73–90 (2012).

[17] A. Klügel, T. H. Hansteen, K. Galipp, Magma storage and underplating beneath Cumbre Vieja volcano, La Palma (Canary Islands). Earth Planet. Sci. Lett. 236, 211–226 (2005).

[18] G. Boudoire, Y-A. Brugier, A. Di Muro, G. Wörner, I. Arienzo, N. Metrich, V. Zanon, N. Braukmüller, A. Kronz, Y. Le Moigne, M. Michon, Eruptive activity on the western flank of Piton de la Fournaise (La Réunion Island, Indian Ocean): Insights on magma transfer, storage and evolution at an oceanic volcanic island. J. Petrol. 60, 1717–1752 (2019).

[19] V. Zanon, M. L. Frezzotti, A. Peccerillo, Magmatic feeding system and crustal magma accumulation beneath Vulcano Island (Italy): Evidence from CO_2_ fluid inclusions in quartz xenoliths. J. Geophys. Res. Solid Earth 108, 2298 (2003).

[20] V. Zanon, I. Nikogosian, Evidence of crustal melting events below the island of Salina (Aeolian arc, southern Italy). Geol. Mag. 141, 525–540 (2004).

[21] V. Zanon, A. Pimentel, M. Auxerre, G. Marchini, F. M. Stuart, Unravelling the magma feeding system of a young basaltic oceanic volcano. Lithos 352-353, 105325 (2020).

[22] V. Zanon, “Conditions for mafic magma storage beneath fissure zones at oceanic islands. The case of São Miguel island (Azores archipelago)” in *Chemical, Physical and Temporal Evolution of Magmatic Systems*, vol. 422, L. Caricchi, J. D. Blundy Eds. (The Geological Society of London, 2015), pp. 85–104.

[23] F. M. Lo Forte, A. Aiuppa, S. G. Rotolo, V. Zanon, Temporal evolution of the fogo volcano magma storage system (cape verde archipelago): A fluid inclusions perspective. J. Volcanol. Geotherm. Res. 433, 107730 (2023).

[24] K. Dayton, E. Gazel, P. Wieser, V. R. Troll, J. C. Carracedo, H. La Madrid, D. C. Roman, J. Ward, M. Aulinas, H. Geiger, F. M. Deegan, G. Gisbert, F. J. Perez-Torrado, Deep magma storage during the 2021 La Palma eruption. Sci. Adv. 9, eade7641 (2023). 36753542 10.1126/sciadv.ade7641 PMC9908012

[25] M. L. Frezzotti, S. Ferrando, F. Tecce, D. Castelli, Water content and nature of solutes in shallow-mantle fluids from fluid inclusions. Earth Planet. Sci. Lett. 351-352, 70–83 (2012).

[26] R. J. Bakker, The perfection of Raman spectroscopic gas densimeters. J. Raman Spectrosc. 52, 1923–1948 (2021).

[27] V. Zanon, K. Cyrzan, L. D'Auria, M. J. Pankhurst, F. Rodríguez, B. Coldwell, A. Martín-Lorenzo, “The magma ascent path during the 2021 eruption of Cumbre Vieja (La Palma Island, Canary archipelago) highlighted by fluid inclusions and seismicity” in *EGU General Assembly Conference Abstracts (Copernicus Publications, 2022)* p. EGU 22-10203. doi: 10.5194/egusphere-egu22-1020.

[28] L. D’Auria, I. Koulakov, J. Prudencio, I. Cabrera-Pérez, J. M. Ibáñez, J. Barrancos, R. García-Hernández, D. M. van Dorth, G. D. Padilla, M. Przeor, V. Ortega, P. Hernández, N. M. Peréz, Rapid magma ascent beneath La Palma revealed by seismic tomography. Sci. Rep. 12, 17654 (2022). 36271131 10.1038/s41598-022-21818-9 PMC9587211

[29] P. A. Torres-González, N. Luengo-Oroz, H. Lamolda, W. D'Alessandro, H. Albert, I. Iribarren, D. Moure-García, V. Soler, Unrest signals after 46 years of quiescence at Cumbre Vieja, La Palma, Canary Islands. J. Volcanol. Geotherm. Res. 392, 106757 (2020).

[30] I. Cabrera-Pérez, L. D'Auria, J. Soubestre, M. Przeor, J. Barrancos, R. García-Hernández, J. M. Ibáñez, I. Koulakov, D. M. van Dorth, V. Ortega, G. D. Padilla, T. Sagiya, N. Pérez, Spatio-temporal velocity variations observed during the pre-eruptive episode of La Palma 2021 eruption inferred from ambient noise interferometry. Sci. Rep. 13, 12039 (2023). 37491500 10.1038/s41598-023-39237-9 PMC10368664

[31] J. E. Romero, M. Burton, F. Cáceres, J. Taddeucci, R. Civico, T. Ricci, M. J. Pankhurst, P. A. Hernández, C. Bonadonna, E. W. Llewellin, M. Pistolesi, M. Polacci, C. Solana, L. D'Auria, F. Arzilli, D. Andronico, F. Rodríguez, M. Asensio-Ramos, A. Martín-Lorenzo, C. Hayera, P. Scarlato, N. M. Perez, The initial phase of the 2021 Cumbre Vieja ridge eruption (Canary Islands): Products and dynamics controlling edifice growth and collapse. J. Volcanol. Geotherm. Res. 431, 107642 (2022).

[32] J. C. Carracedo, V. R. Troll, J. M. D. Day, H. Geiger, M. Aulinas, V. Soler, F. M. Deegan, F. J. Perez-Torrado, G. Gisbert, E. Gazel, A. Rodriguez-Gonzalez, H. Albert, The 2021 eruption of the Cumbre Vieja volcanic ridge on La Palma, Canary Islands. Geol. Today 38, 94–107 (2022).

[33] S. M. Sterner, R. J. Bodnar, Synthetic fluid inclusions - VII. Re-equilibration of fluid inclusions in quartz during laboratory-simulated metamorphic burial and uplift. J. Metamorph. Geol. 7, 243–260 (1989).

[34] M. O. Vityk, R. J. Bodnar, C. S. Schmidt, Fluid inclusions as tectonothermobarometers: Relation between pressure-temperature history and reequilibration morphology during crustal thickening. Geology 22, 731–734 (1994).

[35] M. O. Vityk, R. J. Bodnar, Textural evolution of synthetic fluid inclusions in quartz during reequilibration, with applications to tectonic reconstruction. Contrib. Mineral. Petrol. 121, 309–323 (1995).

[36] V. Zanon, M. L. Frezzotti, Magma storage and ascent conditions beneath Pico and Faial islands (Azores archipelago): A study on fluid inclusions. Geochem. Geophys. Geosystems 14, 3494–3514 (2013).

[37] M. Berkesi, K. Hidas, T. Guzmics, J. Dubessy, R. J. Bodnar, C. Szabo, B. Vajna, T. Tsunogae, Detection of small amounts of H_2_O in CO_2_-rich fluid inclusions using Raman spectroscopy. J Raman Spectrosc. 40, 1461–1463 (2009).

[38] E. Sendula, H. M. Lamadrid, J. D. Rimstidt, M. Steele-MacInnis, D. M. Sublett, L. E. Aradi, C. Szabó, M. J. Caddick, Z. Zajacz, R. J. Bodnar, Synthetic Fluid Inclusions XXIV. In situ monitoring of the carbonation of olivine under conditions relevant to carbon capture and storage using synthetic fluid inclusion micro-reactors: Determination of reaction rates. Front. clim. 3, 722447 (2021).

[39] J. E. Dixon, E. M. Stolper, An experimental study of water and carbon dioxide solubilities in mid-ocean ridge basaltic liquids, Part II: Applications to degassing. J. Petrol. 36, 1633–1646 (1995).

[40] T. H. Hansteen, A. Klügel, Fluid inclusion thermobarometry as a tracer for magmatic processes. Rev. Mineral. Geochem. 69, 143–177 (2008).

[41] T. Andersen, E. A. J. Burke, E. R. Neumann, Nitrogen-rich fluid in the upper mantle: Fluid inclusions in spinel dunite from Lanzarote, Canary Islands. Contrib. Mineral. Petrol. 120, 20–28 (1995).

[42] B. J. Wanamaker, B. Evans, Mechanical re-equilibration of fluid inclusions in San Carlos olivine by power-law creep. Contrib. Mineral. Petrol. 102, 102–111 (1989).

[43] M. O. Vityk, R. J. Bodnar, Statistical microthermometry of synthetic fluid inclusions in quartz during decompression reequilibration. Contrib. Mineral. Petrol. 132, 149–162 (1998).

[44] A. Klügel, K. A. Hoernle, H. U. Schmincke, J. D. White, The chemically zoned 1949 eruption on La Palma (Canary Islands): Petrologic evolution and magma supply dynamics of a rift zone eruption. Geophys. Res. Solid Earth 105, 5997–6016 (2000).

[45] Y. Moussallam, M. A. Longpré, C. McCammon, A. Gomez-Ulla, E. F. Rose-Koga, B. Scaillet, N. Peters, E. Gennaro, R. Paris, C. Oppenheimer, Mantle plumes are oxidised. Earth Planet. Sci. Lett. 527, 115798 (2019).

[46] R. W. Nicklas, R. K. Hahn, L. N. Willhite, M. G. Jackson, V. Zanon, R. Arevalo Jr., J. M. Day, Oxidized mantle sources of HIMU- and EM-type ocean island basalts. Chem. Geol. 602, 120901 (2022).

[47] G. Libourel, B. Marty, F. Humbert, Nitrogen solubility in basaltic melt. Part I. Effect of oxygen fugacity. Geochim. Cosmochim. Acta 67, 4123–4135 (2003).

[48] J. Boulliung, E. Füri, C. Dalou, L. Tissandier, L. Zimmermann, Y. Marrocchi, Oxygen fugacity and melt composition controls on nitrogen solubility in silicate melts. Geochim. Cosmochim. Acta 284, 120–133 (2020).

[49] H. Keppler, L. Cialdella, F. Couffignal, M. Wiedenbeck, The solubility of N_2_ in silicate melts and nitrogen partitioning between upper mantle minerals and basalt. Contrib. Mineral. Petrol. 177, 83 (2022).

[50] A. K. Barker, V. R. Troll, J. C. Carracedo, P. A. Nicholls, The magma plumbing system for the 1971 Teneguía eruption on La Palma, Canary Islands. Contrib. Mineral. Petrol. 170, 54 (2015).

[51] K. Galipp, A. Klügel, T. H. Hansteen, Changing depths of magma fractionation and stagnation during the evolution of an oceanic island volcano: La Palma (Canary Islands). J. Volcanol. Geotherm. Res. 155, 285–306 (2006).

[52] A. Klügel, E. Albers, T. H. Hansteen, Mantle and crustal xenoliths in a tephriphonolite from La Palma (Canary Islands): Implications for phonolite formation at oceanic island volcanoes. Earth Sci. 10, (2022).

[53] M.-A. Longpré, A. Klügel, A. Diehl, J. Stix, Mixing in mantle magma reservoirs prior to and during the 2011–2012 eruption at El Hierro, Canary Islands. Geology 42, 315–318 (2014).

[54] C. Del Fresno, S. Cesca, A. Klügel, I. Domínguez Cerdeña, E. A. Díaz-Suárez, T. Dahm, L. García-Cañada, S. Meletlidis, C. Milkereit, C. Valenzuela-Malebrán, Magmatic plumbing and dynamic evolution of the 2021 La Palma eruption. Communications 14, 358 (2023). 10.1038/s41467-023-35953-y PMC9870893 36690620

[55] A. Klügel, M.-A. Longpré, L. García-Cañada, J. Stix, Deep intrusions, lateral magma transport and related uplift at ocean island volcanoes. Earth Planet. Sci. Lett. 431, 140–149 (2015).

[56] A. Klügel, H.-U. Schmincke, J. D. L. White, K. A. Hoernle, Chronology and volcanology of the 1949 multi-vent rift-zone eruption on La Palma (Canary Islands). J. Volcanol. Geotherm. Res. 94, 267–282 (1999).

[57] C. Szabó, R. J. Bodnar, Changing magma ascent rates in the Nógrád-Gömör volcanic field, northern Hungary/southern Slovakia: Evidence from CO_2_-rich fluid inclusions in metasomatized upper mantle xenoliths. Petrology 4, 221–230 (1996).

[58] F. J. Spera, Carbon dioxide in petrogenesis III: Role of volatiles in the ascent of alkaline magma with special reference to xenolith-bearing mafic lavas. Contrib. Mineral. Petrol. 88, 217–232 (1984).

[59] A. Klügel, Reactions between mantle xenoliths and host magma beneath La Palma (Canary Islands): Constraints on magma ascent rates and crustal reservoirs. Contrib. Mineral. Petrol. 131, 237–257 (1998).

[60] R. T. Helz, C. R. Thornber, Geothermometry of Kilauea Iki lava lake, Hawaii. Bull. Volcanol. 49, 651–668 (1987).

[61] R. Span, W. Wagner, A new equation of state for carbon dioxide covering the fluid region from the triple point temperature to 1100 K at pressures up to 800 MPa. JPCRD 25, 1509–1596 (1996).

[62] S. M. Sterner, K. S. Pitzer, An equation of state for carbon dioxide valid from zero to extreme pressures. Contrib. Mineral. Petrol. 117, 362–374 (1994).

[63] S. M. Sterner, R. J. Bodnar, Synthetic fluid inclusions; X, Experimental determination of P-V-T-X properties in the CO 2 -H 2 O system to 6 kb and 700 degrees C. Am. J. Sci. 291, 1–54 (1991).

[64] R. Thiery, J. Vidal, J. Dubessy, Phase equilibria modelling applied to fluid inclusions: Liquid-vapour equilibria and calculation of the molar volume in the CO_2_ CH_4_ N_2_ system. Geochim. Cosmochim. Acta 58, 1073–1082 (1994).

[65] J. M. Castro, Y. Feisel, Eruption of ultralow-viscosity basanite magma at Cumbre Vieja, La Palma, Canary Islands. Nat. Commun. 13, 3174 (2022). 35676272 10.1038/s41467-022-30905-4 PMC9177865

[66] E. A. J. Burke, Raman microspectrometry of fluid inclusions. Lithos 55, 139–158 (2001).

[67] H. A. Roeser, “Magnetic anomalies in the magnetic quiet zone off Morocco” in *Geology of the Northwest African Continental Margin*, U. Rad, K. Hinz, M. Sarnthein, E. Seibold, Eds. (Springer,1982) pp. 61–68.

[68] J. C. Carracedo, E. R. Badiola, H. Guillou, J. De La Nuez, F. J. Perez Torrado, 2001, Geology and volcanology of La Palma and El Hierro, western Canaries. Estudios Geologicos 57, 175–273 (1982).

[69] K. D. Klitgord, H. Schouten, “Plate kinematics of the Central Atlantic” in *The Geology of North America, v. M: The Western North Atlantic Region*, P. R. Vogt, B. E. Tucholke, Eds. (Geological Society of America, 1986), pp. 351–378.

[70] N.-O. Prægel, P. M. Holm, Lithospheric contributions to high-MgO basanites from the Cumbre Vieja volcano, La Palma, Canary Islands, and evidence for temporal variation in plume influence. J. Volcanol. Geotherm. Res. 149, 213–239 (2006).

[71] K. V. Cashman, R. S. J. Sparks, J. D. Blundy, Vertically extensive and unstable magmatic systems: A unified view of igneous processes. Science 355, (2017). 10.1126/science.aag3055 28336610

[72] R. S. J. Sparks, C. Annen, J. D. Blundy, K. V. Cashman, A. C. Rust, M. D. Jackson, Formation and dynamics of magma reservoirs. Phil. Trans. Royal Soc. A 377, 20180019 (2019). 10.1098/rsta.2018.0019 30966936

[73] A. Lodge, S. E. J. Nippress, A. Rietbrock, A. García-Yeguas, J. M. Ibáñez, Evidence for magmatic underplating and partial melt beneath the Canary Islands derived using teleseismic receiver functions. Phys. Earth Planet. Inter. 212, 44–54 (2012).

[74] C. Martinez-Arevalo, F. de Lis Mancilla, G. Helffrich, A. Garcia, Seismic evidence of a regional sublithospheric low velocity layer beneath the Canary Islands. Tectonophysics 608, 586–599 (2013).

[75] E. Banda, J. J. Dan, E. Surin, J. Ansorge, Features of crustal structure under the Canary Islands. Earth Planet. Sci. Lett. 55, 11–24 (1981).

[76] L. Matias, N. A. Dias, I. Morais, D. Vales, F. Carrilho, J. Madeira, J. L. Gaspar, L. Senos, A. B. Silveira, The 9^th^ of July 1998 Faial Island (Azores, North Atlantic) seismic sequence. J. Seismol. 11, 275–298 (2007).

[77] J. Pim, C. Peirce, A. B. Watts, I. Grevemeyer, A. Krabbenhöft, Crustal structure and origin of the Cape Verde Rise. Earth Planet. Sci. Lett. 272, 422–428 (2008).

[78] C. R. Ranero, M. Torné, E. Banda, Gravity and multichannel seismic reflection constraints on the lithospheric structure of the Canary Swell. Mar. Geophys. Res. 17, 519–534 (1995).

[79] C. J. Lissenberg, C. J. MacLeod, E. N. Bennett, Consequences of a crystal mush-dominated magma plumbing system: A mid-ocean ridge perspective. Phil. Trans. R. Soc. A. 377, 20180014 (2019). 30966931 10.1098/rsta.2018.0014 PMC6335481

[80] K. Benn, A. Nicolas, I. Reuber, Mantle – crust transition zone and origin of wehrlitic magmas: Evidence from the Oman Ophiolite. Tectonophysics 151, 75–85 (1988).

[81] F. Boudier, A. Nicolas, Nature of the Moho transition zone in the Oman ophiolite. J. Petrol. 36, 777–796 (1995).

[82] D. E. James, F. Niu, J. Rokosky, Crustal structure of the Kaapvaal craton and its significance for early crustal evolution. Lithos 71, 413–429 (2003).

[83] B. Ghosh, T. Morishita, B. S. Gupta, A. Tamura, S. Arai, D. Bandyopadhyay, Moho transition zone in the Cretaceous Andaman ophiolite, India: A passage from the mantle to the crust. Lithos 198-199, 117–128 (2014).

[84] J. A. Crisp, Rates of magma emplacement and volcanic output. J. Volcanol. Geotherm. Res. 20, 177–211 (1984).

