# Supplementary material for: Toward a near real-time magma ascent monitoring by combined fluid inclusion barometry and ongoing seismicity
Source: Sci Adv. 2024 Feb 7;10(6):eadi4300. doi: 10.1126/sciadv.adi4300 (PMC10849590; doi:10.1126/sciadv.adi4300)
Supplement: Supplementary file 1 — Supplementary Text Figs. S1 to S4 Tables S1 to S4 References [file sciadv.adi4300_sm.pdf]

Science Advances
AAAS

Supplementary Materials for

Toward a near real-time magma ascent monitoring by combined fluid inclusion barometry and ongoing seismicity

Vittorio Zanon *et al.*

Corresponding author: Vittorio Zanon, vittorio.vz.zanon@azores.gov.pt

Sci. Adv. **10**, eadi4300 (2024)
DOI: 10.1126/sciadv.adi4300

This PDF file includes:

Supplementary Text
Figs. S1 to S4
Table S1 to S4
References

SUPPLEMENTARY MATERIAL

Geological setting

La Palma Island is located on 155 million-year-old oceanic crust (*67-69*) and is composed of the Taburiente and Bejenado structures to the north, which were active for a period lasting from 1.7 million years ago to 410,000 years ago, and the Cumbre Vieja fissure system, which initiated its activity more than 125,000 years ago and makes up the southern portion of the island. The Cumbre Vieja fissure system featured the most active volcanism in the archipelago, erupting along North-South-trending fissures in 1585, 1646, 1677, 1712, 1949, and 1971 (Fig. 1). Its morphology is characterized by rows of cinder cones. Erupted magmas vary from basanite to phonolite (Supplementary Fig. S1A), and recent eruptions have produced products with compositions ranging from tephrite to basanite (*16*, *70*).

Samples and methods

CAN-LLP-0045	lava	25-09-2021	28,60941° N<br>-17,882847° W	Porphyritic lava with clinopyroxenes amphiboles and few olivines
CAN-TLP-0015	tephra	26-09-2021	28,60138° N<br>-17,88822° W	Air fall tephra collected along the road climbing the 1949 lava
CAN-TLP-0022	tephra	29-09-2021	28,60652° N<br>-17,88285° W	Air fall tephra collected along the road heading to El Paraiso from the south
LPA-16	tephra	4-10-2021	28,622904° N<br>-17,873718° W	Air fall tephra collected along the first upper road cut by lava, north side of the cone. With olivines, amphiboles and clinopyroxenes
CAN-TLP-0037	tephra	4-10-2021	28,62533 N<br>-17,88546° W	Air fall tephra along the second upper road cut by lava, north side of the cone. With olivines, amphiboles and clinopyroxenes
CAN-TLP-0039	tephra	5-10-2021	28,60138° N<br>-17,88822° W	Air fall tephra collected from sampling station, containing olivines, clinopyroxenes (rarely green clinopyroxenes)
CAN-TLP-0052	tephra	8-10-2021	28,61712° N<br>-17,84926° W	Air fall tephra from sampling station, with olivines, clinopyroxenes (rarely green clinopyroxene) and very limited plagioclases
CAN-TLP-0053	tephra	8-10-2021	28,61712° N<br>-17,84926° W	Air fall tephra collected from sampling station, containing olivines and clinopyroxenes
CAN-TLP-0128	tephra	19-10-2021	28,60652° N<br>-17,88285° W	Air fall tephra collected from sampling station, containing olivines clinopyroxenes and amphiboles
CAN-LLP-0043	lava	21-10-2021	28,62219° N<br>-17,87368° W	Porphyritic lava with clinopyroxenes and olivines
CAN-LLP-0044	lava	27-10-2021	28,632108° N<br>-17,905858° W	Porphyritic lava with clinopyroxenes and olivines
CAN-LLP-0046	lava	29-10-2021	28,611069° N<br>-17,906714° W	Water-quenched fresh lava containing olivines and clinopyroxenes
CAN-TLP-0214	tephra	6-11-2021	28,60652° N<br>-17,88285° W	Air fall tephra containing olivines and clinopyroxenes
CAN-TLP-0312	tephra	24-11-2021	28,61712° N<br>-17,84926° W	Air fall tephra containing olivines and clinopyroxenes
CAN-TLP-0340	tephra	27–11-2021	28,60652° N<br>-17,88285° W	Air fall tephra containing olivines and clinopyroxenes
CAN-TLP-0370	tephra	2-12-2021	28,60652° N<br>-17,88285° W	Air fall tephra containing olivines and clinopyroxenes
LPA-17	lava	10-12-2021	28,626592° N<br>-17,885083° W	Porphyritic lava with clinopyroxenes and olivines
LPA-18	tephra	10-12-2021	28,617000° N<br>-17,849000° W	Air fall tephra containing clinopyroxenes and olivines

Supplementary table S1 - Main data of studied samples.

	det. lim. wt%	CAN LLP 0043	CAN LLP 0044	CAN LLP 0045	CAN LLP 0046	LPA 17	*NIST 694 Err. %*	*GBW 07113 Err. %*	*SY-4 Err. %*	*BIR 1a Err. %*	*DNC 1a Err. %*	*BCR 2 Err. %*	*W-2b Err. %*
SiO_2 (wt%)	*0.01*	44.61	44.36	43.54	44.26	42.54	*3.57*	*4.81*	*0.81*	*0.77*	*0.92*	*1.23*	*0.82*
TiO_2	*0.001*	3.46	3.413	3.895	3.501	3.493	*3.64*	*7.00*	*0.70*	*2.19*	*1.15*	*1.77*	*2.31*
Al_2O3	*0.01*	14.07	13.83	14.88	13.94	14.24	*3.61*	*2.12*	*2.71*	*1.68*	*1.50*	*0.78*	*1.72*
Fe_2O_3tot	*0.01*	13.52	13.27	13.8	13.49	13.94	*5.06*	*0.31*	*1.53*	*1.42*	*2.11*	*0.54*	*1.21*
MnO	*0.005*	0.184	0.183	0.195	0.182	0.181	*12.93*	*1.07*	*6.02*	*5.71*	*5.33*	*3.06*	*2.15*
MgO	*0.01*	8.41	8.35	6.48	8.15	8.18	*4.55*	*9.38*	*4.63*	*0.52*	*0.64*	*1.11*	*0.63*
CaO	*0.01*	10.97	10.79	10.7	11.09	11.91	*1.79*	*1.69*	*1.49*	*0.56*	*1.57*	*1.76*	*0.09*
Na_2O	*0.01*	3.75	3.73	4.21	3.67	3.73	*0.58*	*3.89*	*2.04*	*0.27*	*1.06*	*4.11*	*3.27*
K_2O	*0.01*	1.47	1.46	1.68	1.42	1.47	*5.88*	*1.01*	*0.90*	*16.67*	*5.98*	*0.28*	*0.96*
P_2O_5	*0.01*	0.75	0.75	1.1	0.75	0.87	*1.11*	*10.00*	*0.76*	*28.57*	*0.00*	*4.29*	*25.00*
LOI		-0.71	-0.4	-0.57	-0.63	-0.34							
**Total**		**100.3**	**99.73**	**99.9**	**99.84**	**100.2**							

Supplementary Table S2 - Detection limits and major element bulk rock compositions of 5 lava samples collected during the eruption. The error (1σ) associated with the analysis of international standards is shown in italics.

	LPA-16 *N=11*	*Std dev*	CAN-TLP-0053 *N=10*	*Std dev*	CAN-TLP-0128#1 *N=5*	*Std dev*	CAN-TLP-0128#2 *N=5*	*Std dev*	CAN-TLP-0128#3 *N=5*	*Std dev*	***ALV N=18***	*Std dev*	***CH98-DR11 N=18***	*Std dev*
**SiO_2 (wt%)**	46.02	*1.38*	46.09	*1.00*	46.41	*1.44*	47.13	*1.88*	45.15	*0.11*	*48.01*	*0.30*	*48.89*	*0.87*
**TiO_2**	3.46	*0.70*	3.56	*0.38*	3.32	*0.77*	2.98	*1.10*	3.95	*0.01*	*1.29*	*0.05*	*1.50*	*0.05*
**Al_2O3**	17.38	*2.65*	16.99	*1.47*	18.63	*3.23*	20.07	*4.48*	15.99	*0.13*	*16.24*	*0.16*	*14.64*	*0.22*
**FeO**	10.03	*2.00*	10.55	*1.09*	9.57	*2.36*	9.40	*2.90*	11.81	*0.29*	*8.83*	*0.18*	*10.19*	*0.11*
**MnO**	0.18	*0.04*	0.24	*0.05*	0.23	*0.02*	0.21	*0.02*	0.22	*0.03*	*0.18*	*0.03*	*0.20*	*0.03*
**MgO**	3.30	*0.75*	3.39	*0.43*	3.12	*0.83*	3.00	*1.23*	3.92	*0.04*	*8.47*	*0.09*	*8.31*	*0.14*
**CaO**	9.19	*0.65*	8.99	*0.36*	9.95	*0.87*	9.99	*0.82*	9.29	*0.15*	*11.75*	*0.07*	*11.03*	*0.16*
**Na_2O**	5.40	*0.31*	5.46	*0.24*	4.95	*0.35*	4.81	*0.45*	5.31	*0.12*	*2.88*	*0.07*	*2.56*	*0.07*
**K_2O**	2.32	*0.42*	2.45	*0.32*	1.97	*0.52*	1.83	*0.66*	2.35	*0.06*	*0.08*	*0.02*	*0.09*	*0.01*
**P_2O_5**	1.29	*0.25*	1.36	*0.14*	1.09	*0.22*	0.98	*0.34*	1.25	*0.02*	*0.09*	*0.01*	*0.09*	*0.01*
**SO_2**	0.08	*0.03*	0.09	*0.03*	0.09	*0.03*	0.07	*0.02*	0.08	*0.03*	*0.22*	*0.03*	*0.25*	*0.02*
**Cl**	0.09	*0.02*	0.09	*0.02*	0.09	*0.01*	0.11	*0.01*	0.09	*0.01*	*0.22*	*0.04*	*0.23*	*0.04*
**Total**	98.75		99.25		99.42		100.57		99.42		*98.24*		*97.98*	
**T (°C)**	1081		1084		1076		1073		1097					

Supplementary Table S3 - Electron probe data from 5 glassy shards from early tephra samples used to calculate eruption temperature. N is the number of spots per shard used in the average. In italics are reported analyses of two international glass standards.

Reference stratigraphy model

The reference stratigraphic model below the Cumbre Vieja volcano, shown in Supplementary Figure 3, is used to calculate depths from FI-derived pressures. It is constructed by combining the quantitative information on v_p/v_s, the depth of earthquakes (*27*), and textural and barometric information from fluid inclusions, and is consistent with the new paradigm of the magma system (*71*, *72*). A key issue is the location of the crust-mantle boundary, as it defines the separation of lower-crust rocks with densities of ~2800 $kg \cdot m^{-3}$ from mantle lithologies characterized by densities of ~3100-3400 $kg \cdot m^{-3}$. This depth is not only fundamental for correct recalculation of pressures from FI, but also allows precise localization of seismic events in XYZ space (*20*). Geophysical studies locate the crust-mantle transition where a major abrupt jump in seismic wave velocities (v_p/v_s) is observed. This depth has been identified at ~12 to 15 km beneath the island of La Palma (*73*, *74*). Interestingly, this is the same depth range found under the other oceanic islands of the archipelago and the Atlantic Ocean (i.e., Azores, Cape Verde, Madeira; *75-78*). This is anomalous because this depth should increase with distance from the Mid-Atlantic Ridge and with the age of a volcanic island.

On oceanic volcanic islands, the amount of melt ascending through the crust is relatively small and preferentially accumulated at its base, and cooling is so efficient that a steady-state melt lens cannot be maintained (*79*). Melt crystallization promotes the formation of a number of plutonic bodies and ultramafic cumulates, and thus the progressive accumulation of unerupted magmas contributes to the generation of new crust, which is geophysically indistinguishable from the mantle due to its mineralogical composition and is interbedded with portions of the lithospheric mantle. The true boundary between the crust and the lithospheric mantle is therefore elusive and is distributed in a transition zone of variable thickness (e.g., *80-83*).

In this framework, plutonic bodies and ultramafic cumulates with different mineralogies define a complex puzzle for geophysicists who have to interpret multiple jumps in v_p/v_s. The deepest ultramafic cumulates measured present rock density values closer to those canonical of the lithospheric mantle. As a result, we suggest that the most significant jump in v_p/v_s represents the transition from Jurassic MORB lavas to an intrusive/cumulate complex formed by prolonged and focused magmatism (rather than a sharp crust-mantle boundary). It is here hypothesized that the roots of the Cumbre Vieja volcano could be found after a ~5 km thick a-seismic zone that separates the two main seismic regions beneath the Tajogaite volcano (*27*). Crystal mush layers interbedded with lenses or layers of mantle lithologies may form the deep roots of this volcano, starting at ~20 km depth.

Time-integrated intruded magma volumes

Estimates of intruded vs. extruded magmas range from 3:1 to 5:1 on a global scale (*84*). If we assume that:

- 0.2 km^3 of magma erupted during the 2021 eruption represents the volume erupted during one century (conservative hypothesis);

- the eruption rate is constant throughout the volcanic history of Cumbre Vieja;

- the minimum age of the subaerial part of the Cumbre Vieja volcano corresponds to the highest available dates (>123,000 years; *67*);

- the ascending magmas stop at the crust-mantle transition, distributing along a volume similar to an ellipsoid;

- two radii of the ellipsoid can be represented by the 3D distribution of earthquakes during the 2021 eruption and by half the length of the volcano's fissure system (i.e., 7 and 9 km);

- the Jurassic oceanic crust and overlying sediments have a total thickness of 7 km (conservative estimate);

- the presence of pre-existing intrusive layers from the activity of older volcanic edifices is neglected (conservative estimate),

then we can calculate conservative values of 16.7 and 20.4 km for the present thickness of the crust from sea level, which are deeper than those calculated by geophysicists. These values do not agree with the observed seismicity and fluid inclusion data.

**Age (years)**	**intruded volume (km^3)**	**Ellipsoid height (km)**	**crust-mantle transition depth (km)**
123000	*3:1* 738	5.6	16.7
	5:1 1230	9.3	20.4
200000	*3:1* 1200	9.1	20.2
	5:1 2000	15.2	26.3
250000	*3:1* 1500	11.4	22.5
	5:1 2500	19.0	30.1
275000	*3:1* 1650	12.5	23.6
	5:1 2750	20.9	32.0
300000	*3:1* 1800	13.6	30.4
	5:1 3000	22.7	43.3

Supplementary table S4 – simulation of the crust-mantle transition depth range for different ages of volcanic activity.

If geobarometric data from FI and deep seismicity highlight the main magma accumulation zone beneath the volcano at depths of 20 to 27 km, then a volcanic history of ~200 to ~250 ka would be required to reach this thickness.

Supplementary figures

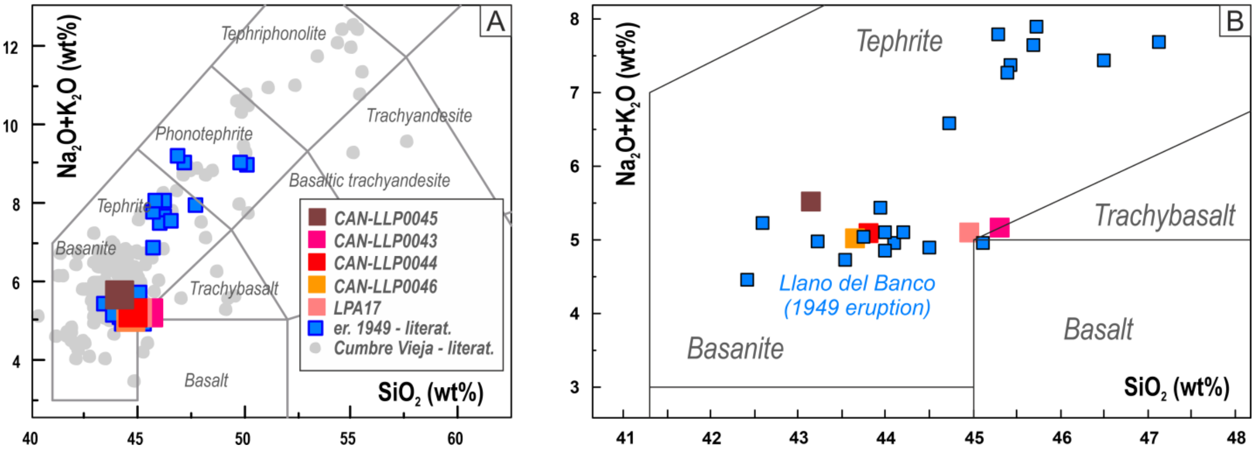

Supplementary Figure S1 - Classification of the erupted rocks. (A) Total alkali-silica diagram showing the composition of the lava samples studied and compared with the erupted products of Cumbre Vieja from the literature (Georoc database). (B) Detail of the basanite and alkali-basalt fields, showing the composition of rocks erupted during the 1949 eruption for comparison (*43*).

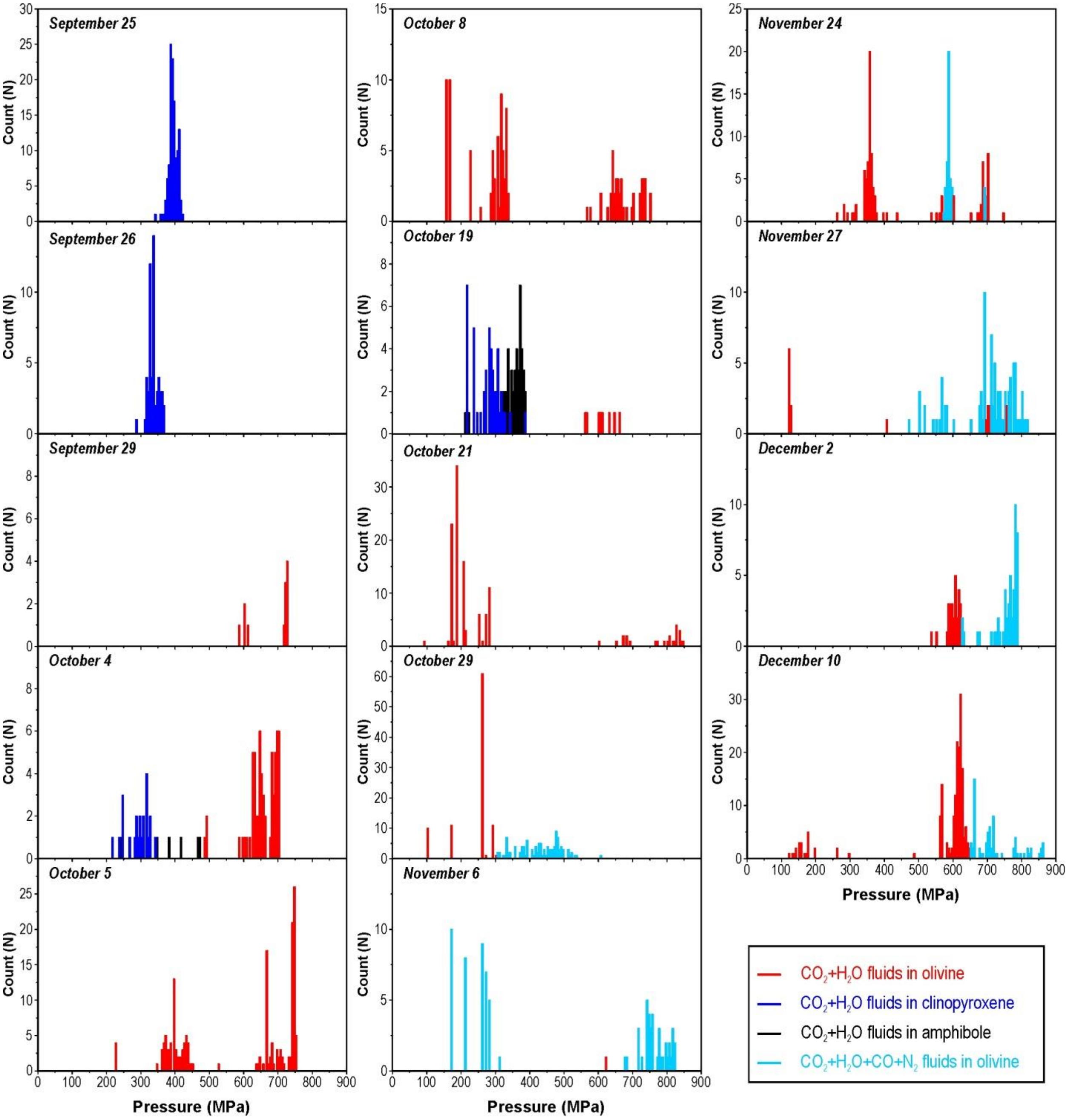

Supplementary Figure S2 - Histograms of barometric data from fluid inclusions microthermometry. Blue, black and red bars represent CO_2+H_2O data from clinopyroxene, amphibole and olivine, respectively. Cyan represents CO_2+H_2O+CO+N_2 fluid inclusions in olivine. Model eruption temperatures used are 1075 °C for FI in amphiboles and clinopyroxenes, and 1150 °C for FI in olivines.

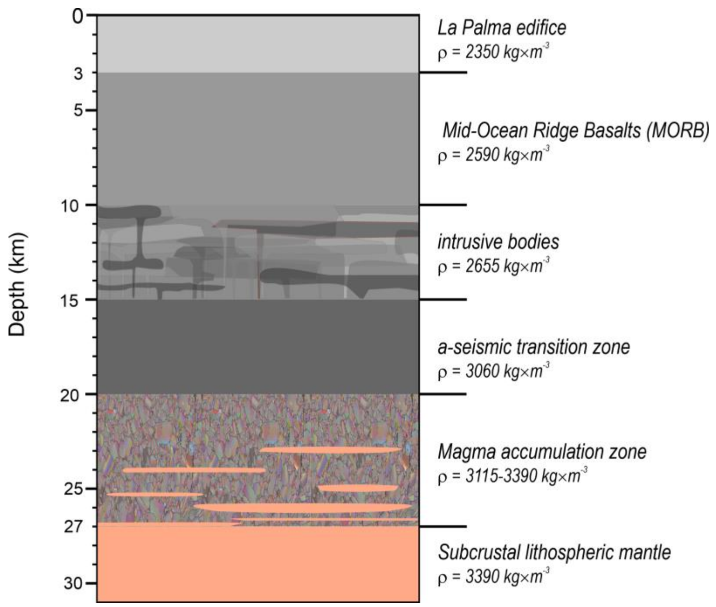

Supplementary Figure S3 - Reference conceptual stratigraphic model. The upper layer (~3 km) corresponds to the island edifice made of lavas overlying the oceanic crust of basaltic composition (down to depths of 10 km). Intrusive bodies and mafic cumulates are located down to depths of 15 km, followed by a sequence of mafic rocks that form the deep magma feeding system. Rock density values have been assigned to shallow lavas, dense gabbroic xenoliths and mantle lithologies after measurements with an electronic densimeter, and assumed for the other rock bodies.

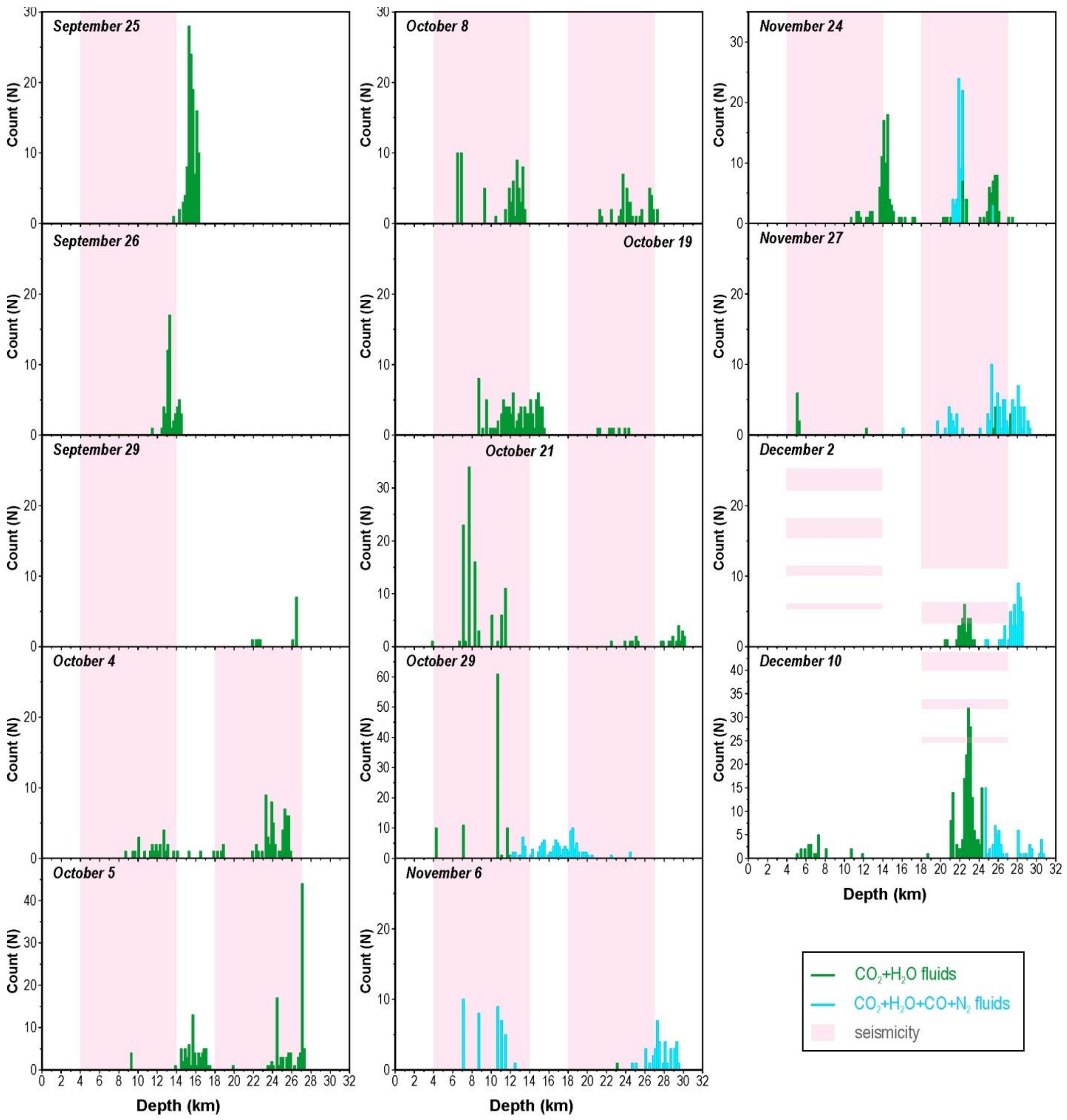

Supplementary Figure S4 - Histograms of depth of trapping/re-equilibration of FI and recorded seismicity. Green bars represent pure CO_2 (+H_2O) FI (hosted in clinopyroxene, amphibole and olivine) and cyan bars represent olivine-hosted N_2 (±CO)-bearing FI. Seismicity is represented by pale pink stripes according to (*27*). These stripes, when dashed, indicate decreasing seismicity.

REFERENCES AND NOTES

1. A. García, A. Fernández-Ros, M. Berrocoso, J. M. Marrero, G. Prates, S. De la Cruz-Reyna, R. Ortiz, Magma displacements under insular volcanic fields, applications to eruption forecasting: El Hierro, Canary Islands, 2011–2013. *Geophys. J. Int.* **197**, 322–334 (2014).

2. Z. Duputel, O. Lengliné, V. Ferrazzini, Constraining spatiotemporal characteristics of magma migration at Piton de la Fournaise volcano from pre-eruptive seismicity. *Geophys. Res. Lett.* **46**, 119–127 (2019).

3. M. J. Pankhurst, D. J. Morgan, T. Thordarson, S. C. Loughlin, Magmatic crystal records in time, space, and process, causatively linked with volcanic unrest. *Earth Planet. Sci. Lett.* **493**, 231–241 (2018).

4. D. J. Rasmussen, T. A. Plank, D. C. Roman, J. A. Power, R. J. Bodnar, E. H. Hauri, When does eruption run-up begin? Multidisciplinary insight from the 1999 eruption of Shishaldin volcano. *Earth Planet. Sci. Lett.* **486**, 1–14 (2018).

5. T. Ubide, Á. Márquez, E. Ancochea, M. J. Huertas, R. Herrera, J. J. Coello-Bravo, D. Sanz-Mangas, J. Mulder, A. MacDonald, I. Galindo, Discrete magma injections drive the 2021 La Palma eruption. *Sci. Adv.* **9**, eadg4813 (2023).

6. R. Corsaro, L. Miraglia, V. Zanon, Petrologic monitoring of glasses in the pyroclastites erupted in February 2004 by the Stromboli Volcano, Aeolian Islands, Southern Italy, *J. Volcanol. Geotherm. Res.* **139**, 339–343 (2005).

7. S. A. Halldórsson, E. Bali, M. E. Hartley, D. A. Neave, D. W. Peate, G. H. Guðfinnsson, I. Bindeman, M. J. Whitehouse, M. S. Riishuus, G. B. M. Pedersen, S. Jakobsson, R. Askew, C. R. Gallagher, E. R. Guðmundsdóttir, J. Gudnason, W. M. Moreland, B. W. Óskarsson, P. Nikkola, H. I. Reynolds, J. Schmith, T. Thordarson, Petrology and geochemistry of the 2014–2015 Holuhraun eruption, central Iceland: Compositional and mineralogical characteristics, temporal variability and magma storage. *Contrib. Mineral. Petrol.* **173**, 1–25 (2018).

8. P. Landi, R. A. Corsaro, L. Francalanci, L. Civetta, L. Miraglia, M. Pompilio, R. Tesoro, Magma dynamics during the 2007 Stromboli eruption (Aeolian Islands, Italy): Mineralogical, geochemical and isotopic data. *J. Volcanol. Geotherm. Res.* **182**, 255–268 (2009).

9. N. Spilliaert, P. Allard, N. Métrich, A. V. Sobolev, Melt inclusion record of the conditions of ascent, degassing, and extrusion of volatile-rich alkali basalt during the powerful 2002 flank eruption of Mount Etna (Italy), *J. Geophys. Res. Solid* **111**, B04203 (2006).

10. C. Gansecki, R. L. Lee, T. Shea, S. P. Lundblad, K. Hon, C. Parcheta, The tangled tale of Kīlauea's 2018 eruption as told by geochemical monitoring. *Science* **366**, eaaz0147 (2019).

11. S. A. Halldórsson, E. W. Marshall, A. Caracciolo, S. Matthews, E. Bali, M. B. Rasmussen, E. Ranta, J. Gunnarsson-Robin, G. H. Guðfinnsson, O. Sigmarsson, J. Maclennan, M. G. Jackson, M. J. Whitehouse, H. Jeon, Q. H. A. van der Meer, G. K. Mibei, M. H. Kalliokoski, M. M. Repczynska, R. H. Rúnarsdóttir, G. Sigurðsson, M. A. Pfeffer, S. W. Scott, R. Kjartansdóttir, B. I. Kleine, C. Oppenheimer, A. Aiuppa, E. Ilyinskaya, M. Bitetto, G. Giudice, A. Stefánsson, Rapid shifting of a deep magmatic source at Fagradalsfjall volcano, Iceland, *Nature* **609**, 529–534 (2022).

12. J. M. Day, V. R. Troll, M. Aulinas, F. M. Deegan, H. Geiger, J. C. Carracedo, G. Gisbert Pinto, F. J. Perez-Torrado, Mantle source characteristics and magmatic processes during the 2021 La Palma eruption. *Earth Planet. Sci. Lett.* **597**, 117793 (2022).

13. K. D. Putirka, "Thermometers and barometers for volcanic systems" in *Minerals, Inclusions and Volcanic Processes*, vol. 69 of Reviews in Mineralogy Geochemistry, K. D. Putirka, F. Tepley, Eds. (Mineralogical Society of America, 2008), pp. 61–120.

14. P. E. Wieser, A. J. F. Kent, C. B. Till, J. Donovan, D. A. Neave, D. L. Blatter, M. J. Krawczynski, Barometers behaving badly: Assessing the influence of analytical and experimental uncertainty on clinopyroxene thermobarometry calculations at crustal conditions. *J. Petrol.* **64**, egac126 (2022).

15. T. H. Hansteen, A. Klügel, H.-U. Schmincke, Multi-stage magma ascent beneath the Canary Islands: Evidence from fluid inclusions. *Contrib. Mineral. Petrol.* **132**, 48–64 (1998).

16. E. Hildner, A. Klügel, T. H. Hansteen, Barometry of lavas from the 1951 eruption of Fogo, Cape Verde Islands: Implications for historic and prehistoric magma plumbing systems. *J. Volcanol. Geotherm. Res.* **217–218**, 73–90 (2012).

17. A. Klügel, T. H. Hansteen, K. Galipp, Magma storage and underplating beneath Cumbre Vieja volcano, La Palma (Canary Islands). *Earth Planet. Sci. Lett.* **236**, 211–226 (2005).

18. G. Boudoire, Y-A. Brugier, A. Di Muro, G. Wörner, I. Arienzo, N. Metrich, V. Zanon, N. Braukmüller, A. Kronz, Y. Le Moigne, M. Michon, Eruptive activity on the western flank of Piton de la Fournaise (La Réunion Island, Indian Ocean): Insights on magma transfer, storage and evolution at an oceanic volcanic island. *J. Petrol.* **60**, 1717–1752 (2019).

19. V. Zanon, M. L. Frezzotti, A. Peccerillo, Magmatic feeding system and crustal magma accumulation beneath Vulcano Island (Italy): Evidence from CO_2 fluid inclusions in quartz xenoliths. *J. Geophys. Res. Solid Earth* **108**, 2298 (2003).

20. V. Zanon, I. Nikogosian, Evidence of crustal melting events below the island of Salina (Aeolian arc, southern Italy). *Geol. Mag.* **141**, 525–540 (2004).

21. V. Zanon, A. Pimentel, M. Auxerre, G. Marchini, F. M. Stuart, Unravelling the magma feeding system of a young basaltic oceanic volcano. *Lithos* **352-353**, 105325 (2020).

22. V. Zanon, "Conditions for mafic magma storage beneath fissure zones at oceanic islands. The case of São Miguel island (Azores archipelago)" in *Chemical, Physical and Temporal Evolution of Magmatic Systems*, vol. 422, L. Caricchi, J. D. Blundy Eds. (The Geological Society of London, 2015), pp. 85–104.

23. F. M. Lo Forte, A. Aiuppa, S. G. Rotolo, V. Zanon, Temporal evolution of the fogo volcano magma storage system (cape verde archipelago): A fluid inclusions perspective. *J. Volcanol. Geotherm. Res.* **433**, 107730 (2023).

24. K. Dayton, E. Gazel, P. Wieser, V. R. Troll, J. C. Carracedo, H. La Madrid, D. C. Roman, J. Ward, M. Aulinas, H. Geiger, F. M. Deegan, G. Gisbert, F. J. Perez-Torrado, Deep magma storage during the 2021 La Palma eruption. *Sci. Adv.* **9**, eade7641. (2023).

25. M. L. Frezzotti, S. Ferrando, F. Tecce, D. Castelli, Water content and nature of solutes in shallow-mantle fluids from fluid inclusions. *Earth Planet. Sci. Lett.* **351-352**, 70–83 (2012).

26. R. J. Bakker, The perfection of Raman spectroscopic gas densimeters, *J. Raman Spectrosc.* **52**, 1923–1948 (2021).

27. V. Zanon, K. Cyrzan, L. D'Auria, M. J. Pankhurst, F. Rodríguez, B. Coldwell, A. Martín-Lorenzo, "The magma ascent path during the 2021 eruption of Cumbre Vieja (La Palma Island, Canary archipelago) highlighted by fluid inclusions and seismicity" in *EGU General Assembly Conference Abstracts* (Copernicus Publications, 2022) p. EGU22-10203. doi:10.5194/egusphere-egu22-1020 (2022).

28. L. D'Auria, I. Koulakov, J. Prudencio, I. Cabrera-Pérez, J. M. Ibáñez, J. Barrancos, R. García-Hernández, D. M. van Dorth, G. D. Padilla, M. Przeor, V. Ortega, P. Hernández, N. M. Peréz, Rapid magma ascent beneath La Palma revealed by seismic tomography. *Sci. Rep.* **12**, 17654 (2022).

29. P. A. Torres-González, N. Luengo-Oroz, H. Lamolda, W. D'Alessandro, H. Albert, I. Iribarren, D. Moure-García, V. Soler, Unrest signals after 46 years of quiescence at Cumbre Vieja, La Palma, Canary Islands, *J. Volcanol. Geotherm. Res.* **392**, 106757 (2020).

30. I. Cabrera-Pérez, L. D'Auria, J. Soubestre, M. Przeor, J. Barrancos, R. García-Hernández, J. M. Ibáñez, I. Koulakov, David Martínez van Dorth, V. Ortega, G. D. Padilla, T. Sagiya, N. Pérez, Spatio-temporal velocity variations observed during the pre-eruptive episode of La Palma 2021 eruption inferred from ambient noise interferometry. *Sci. Rep.* **13**, 12039 (2023).

31. J. E. Romero, M. Burton, F. Cáceres, J. Taddeucci, R. Civico, T. Ricci, M. J. Pankhurst, P. A. Hernández, C. Bonadonna, E. W. Llewellin, M. Pistolesi, M. Polacci, C. Solana, L. D'Auria, F. Arzilli, D. Andronico, F. Rodríguez, M. Asensio-Ramos, A. Martín-Lorenzo, C. Hayera, P. Scarlato, N. M. Perez, The initial phase of the 2021 Cumbre Vieja ridge eruption (Canary Islands): Products and dynamics controlling edifice growth and collapse. *J. Volcanol. Geotherm. Res.* **431**, 107642 (2022).

32. J. C. Carracedo, V. R. Troll, J. M. D. Day, H. Geiger, M. Aulinas, V. Soler, F. M. Deegan, F. J. Perez-Torrado, G. Gisbert, E. Gazel, A. Rodriguez-Gonzalez, H. Albert, The 2021 eruption of the Cumbre Vieja volcanic ridge on La Palma, Canary Islands, *Geol. Today*, **38**, 94–107 (2022).

33. S. M. Sterner, R. J. Bodnar, Synthetic fluid inclusions - VII. Re-equilibration of fluid inclusions in quartz during laboratory-simulated metamorphic burial and uplift. *J. Metamorph. Geol.* **7**, 243–260 (1989).

34. M. O. Vityk, R. J. Bodnar, C. S. Schmidt, Fluid inclusions as tectonothermobarometers: Relation between pressure-temperature history and reequilibration morphology during crustal thickening. *Geology* **22**, 731–734 (1994).

35. M. O. Vityk, R. J. Bodnar, Textural evolution of synthetic fluid inclusions in quartz during reequilibration, with applications to tectonic reconstruction. *Contrib. Mineral. Petrol.* **121**, 309–323 (1995).

36. V. Zanon, M. L. Frezzotti, Magma storage and ascent conditions beneath Pico and Faial islands (Azores archipelago): A study on fluid inclusions. *Geochem. Geophys. Geosystems* **14**, 3494–3514 (2013).

37. M. Berkesi, K. Hidas, T. Guzmics, J. Dubessy, R. J. Bodnar, C. Szabo, B. Vajna, T. Tsunogae, Detection of small amounts of H_2O in CO_2-rich fluid inclusions using Raman spectroscopy. *J Raman Spectrosc.* **40**, 1461–1463 (2009).

38. E. Sendula, H. M. Lamadrid, J. D. Rimstidt, M. Steele-MacInnis, D. M. Sublett, L. E. Aradi, C. Szabó, M. J. Caddick, Z. Zajacz, R. J. Bodnar, Synthetic Fluid Inclusions XXIV. In situ monitoring of the carbonation of olivine under conditions relevant to carbon capture and storage using synthetic fluid inclusion micro-reactors: Determination of reaction rates. *Front. clim.* **3**, 722447 (2021).

39. J. E. Dixon, E. M. Stolper, An experimental study of water and carbon dioxide solubilities in mid-ocean ridge basaltic liquids, Part II: Applications to degassing. *J. Petrol.* **36**, 1633–1646 (1995).

40. T. H. Hansteen, A. Klügel, Fluid inclusion thermobarometry as a tracer for magmatic processes *Rev. Mineral. Geochem.* **69**, 143–177 (2008).

41. T. Andersen, E. A. J. Burke, E. R. Neumann, Nitrogen-rich fluid in the upper mantle: Fluid inclusions in spinel dunite from Lanzarote, Canary Islands. *Contrib. Mineral. Petrol.* **120**, 20–28 (1995).

42. B. J. Wanamaker, B. Evans, Mechanical re-equilibration of fluid inclusions in San Carlos olivine by power-law creep. *Contrib. Mineral. Petrol.* **102**, 102–111 (1989).

43. M. O. Vityk, R. J. Bodnar, Statistical microthermometry of synthetic fluid inclusions in quartz during decompression reequilibration. *Contrib. Mineral. Petrol.* **132**, 149–162 (1998).

44. A. Klügel, K. A. Hoernle, H. U. Schmincke, J. D. White, The chemically zoned 1949 eruption on La Palma (Canary Islands): Petrologic evolution and magma supply dynamics of a rift zone eruption. *Geophys. Res. Solid Earth* **105**, 5997–6016 (2000).

45. Y. Moussallam, M. A. Longpré, C. McCammon, A. Gomez-Ulla, E. F. Rose-Koga, B. Scaillet, N. Peters, E. Gennaro, R. Paris, C. Oppenheimer, Mantle plumes are oxidised. *Earth Planet. Sci. Lett.* **527**, 115798 (2019).

46. R. W. Nicklas, R. K. Hahn, L. N. Willhite, M. G. Jackson, V. Zanon, R. Arevalo Jr, J. M. Day, Oxidized mantle sources of HIMU- and EM-type ocean island basalts. *Chem. Geol.* **602**, 120901 (2022).

47. G. Libourel, B. Marty, F. Humbert, Nitrogen solubility in basaltic melt. Part I. Effect of oxygen fugacity. *Geochim. Cosmochim. Acta* **67**, 4123–4135 (2003).

48. J. Boulliung, E. Füri, C. Dalou, L. Tissandier, L. Zimmermann, Y. Marrocchi, Oxygen fugacity and melt composition controls on nitrogen solubility in silicate melts. *Geochim. Cosmochim. Acta* **284**, 120–133 (2020).

49. H. Keppler, L. Cialdella, F. Couffignal, M. Wiedenbeck, The solubility of N_2 in silicate melts and nitrogen partitioning between upper mantle minerals and basalt. *Contrib. Mineral. Petrol.* **177**, 83 (2022).

50. A. K. Barker, V. R. Troll, J. C. Carracedo, P. A. Nicholls, The magma plumbing system for the 1971 Teneguía eruption on La Palma, Canary Islands, *Contrib. Mineral. Petrol.* **170**, 54 (2015).

51. K. Galipp, A. Klügel, T. H. Hansteen, Changing depths of magma fractionation and stagnation during the evolution of an oceanic island volcano: La Palma (Canary Islands). *J. Volcanol. Geotherm. Res.* **155**, 285–306 (2006).

52. A. Klügel, E. Albers, T. H. Hansteen, Mantle and crustal xenoliths in a tephriphonolite from La Palma (Canary Islands): Implications for phonolite formation at oceanic island volcanoes. *Earth Sci.* **10** (2022).

53. M.-A. Longpré, A. Klügel, A. Diehl, J. Stix, Mixing in mantle magma reservoirs prior to and during the 2011–2012 eruption at El Hierro, Canary Islands *Geology* **42**, 315–318 (2014).

54. C. Del Fresno, S. Cesca, A. Klügel, I. Domínguez Cerdeña, E. A. Díaz-Suárez, T. Dahm, L. García-Cañada, S. Meletlidis, C. Milkereit, C. Valenzuela-Malebrán, Magmatic plumbing and dynamic evolution of the 2021 La Palma eruption *Communications* **14**, 358 (2023).

55. A. Klügel, M.-A. Longpré, L. García-Cañada, J. Stix, Deep intrusions, lateral magma transport and related uplift at ocean island volcanoes. *Earth Planet. Sci. Lett.***431**, 140–149 (2015).

56. A. Klügel, H.-U. Schmincke, J. D. L. White, K. A. Hoernle, Chronology and volcanology of the 1949 multi-vent rift-zone eruption on La Palma (Canary Islands). *J. Volcanol. Geotherm. Res.* **94**, 267–282 (1999).

57. C. Szabó, R. J. Bodnar, Changing magma ascent rates in the Nógrád-Gömör volcanic field, northern Hungary/southern Slovakia: Evidence from CO_2-rich fluid inclusions in metasomatized upper mantle xenoliths. *Petrology* **4**, 221–230 (1996).

58. F. J. Spera, Carbon dioxide in petrogenesis III: Role of volatiles in the ascent of alkaline magma with special reference to xenolith-bearing mafic lavas. *Contrib. Mineral. Petrol.* **88**, 217–232 (1984).

59. A. Klügel, Reactions between mantle xenoliths and host magma beneath La Palma (Canary Islands): Constraints on magma ascent rates and crustal reservoirs. *Contrib. Mineral. Petrol.* **131**, 237–257. (1998).

60. R. T. Helz, C. R. Thornber, Geothermometry of Kilauea Iki lava lake, Hawaii, *Bull. Volcanol.* **49**, 651–668 (1987).

61. R. Span, W. Wagner, A new equation of state for carbon dioxide covering the fluid region from the triple point temperature to 1100 K at pressures up to 800 MPa, *JPCRD* **25**, 1509–1596 (1996).

62. S. M. Sterner, K. S. Pitzer, An equation of state for carbon dioxide valid from zero to extreme pressures. *Contrib. Mineral. Petrol.* **117**, 362–374 (1994).

63. S. M. Sterner, R. J. Bodnar, Synthetic fluid inclusions; X, Experimental determination of P-V-T-X properties in the CO 2 -H 2 O system to 6 kb and 700 degrees C, *Am. J. Sci.*, **291**, 1–54 (1991).

64. R. Thiery, J. Vidal, J. Dubessy, Phase equilibria modelling applied to fluid inclusions: Liquid-vapour equilibria and calculation of the molar volume in the CO_2 CH_4 N_2 system. *Geochim. Cosmochim. Acta* **58**, 1073–1082 (1994).

65. J. M. Castro, Y. Feisel, Eruption of ultralow-viscosity basanite magma at Cumbre Vieja, La Palma, Canary Islands, *Nat. Commun.* **13**, 3174, (2022).

66. E. A. J. Burke, Raman microspectrometry of fluid inclusions. *Lithos* **55**, 139–158 (2001).

67. H. A. Roeser, "Magnetic anomalies in the magnetic quiet zone off Morocco" in *Geology of the Northwest African Continental Margin*, U. Rad, K. Hinz, M. Sarnthein, E. Seibold, Eds. (Springer,1982) pp. 61–68.

68. J. C. Carracedo, E. R. Badiola, H. Guillou, J. De La Nuez, F. J. Perez Torrado, 2001, Geology and volcanology of La Palma and El Hierro, western Canaries. *Estudios Geologicos* **57**, 175–273 (1982).

69. K. D. Klitgord, H. Schouten, “Plate kinematics of the Central Atlantic” in *The Geology of North America, v. M: The Western North Atlantic Region*, P. R. Vogt, B. E. Tucholke, Eds. (Geological Society of America, 1986), pp. 351–378.

70. N.-O. Prægel, P. M. Holm, Lithospheric contributions to high-MgO basanites from the Cumbre Vieja volcano, La Palma, Canary Islands, and evidence for temporal variation in plume influence. *J. Volcanol. Geotherm. Res.* **149**, 213–239 (2006).

71. K. V. Cashman, R. S. J. Sparks, J. D. Blundy, Vertically extensive and unstable magmatic systems: A unified view of igneous processes. *Science* **355** (2017).

72. R. S. J. Sparks, C. Annen, J. D. Blundy, K. V. Cashman, A. C. Rust, M. D. Jackson, Formation and dynamics of magma reservoirs. *Phil. Trans. Royal Soc. A* **377**, 20180019 (2019).

73. A. Lodge, S. E. J. Nippress, A. Rietbrock, A. García-Yeguas, J. M. Ibáñez, Evidence for magmatic underplating and partial melt beneath the Canary Islands derived using teleseismic receiver functions. *Phys. Earth Planet. Inter.* **212**, 44–54 (2012).

74. C. Martinez-Arevalo, F. de Lis Mancilla, G. Helffrich, A. Garcia, Seismic evidence of a regional sublithospheric low velocity layer beneath the Canary Islands. *Tectonophysics* **608**, 586–599 (2013).

75. E. Banda, J. J. Dan, E. Surin, J. Ansorge, Features of crustal structure under the Canary Islands. *Earth Planet. Sci. Lett.* **55**,11–24(1981).

76. L. Matias, N. A. Dias, I. Morais, D. Vales, F. Carrilho, J. Madeira, J. L. Gaspar, L. Senos, A. B. Silveira, The 9th of July 1998 Faial Island (Azores, North Atlantic) seismic sequence. *J. Seismol.* **11**, 275–298 (2007).

77. J. Pim, C. Peirce, A. B. Watts, I. Grevemeyer, A. Krabbenhöft, Crustal structure and origin of the Cape Verde Rise. *Earth Planet. Sci. Lett.* **272**, 422–428 (2008).

78. C. R. Ranero, M. Torné, E. Banda, Gravity and multichannel seismic reflection constraints on the lithospheric structure of the Canary Swell. *Mar. Geophys. Res.* **17**, 519–534 (1995).

79. C. J. Lissenberg, C. J. MacLeod, E. N. Bennett, Consequences of a crystal mush-dominated magma plumbing system: A mid-ocean ridge perspective. *Phil. Trans. R. Soc. A.* **377**, 20180014 (2019).

80. K. Benn, A. Nicolas, I. Reuber, Mantle – crust transition zone and origin of wehrlitic magmas: Evidence from the Oman Ophiolite. *Tectonophysics* **151**, 75–85 (1988).

81. F. Boudier, A. Nicolas, Nature of the Moho transition zone in the Oman ophiolite. *J. Petrol.* **36**, 777–796 (1995).

82. D. E. James, F. Niu, J. Rokosky, Crustal structure of the Kaapvaal craton and its significance for early crustal evolution. *Lithos* **71**, 413–429 (2003).

83. B. Ghosh, T. Morishita, B. S. Gupta, A. Tamura, S. Arai, D. Bandyopadhyay, Moho transition zone in the Cretaceous Andaman ophiolite, India: A passage from the mantle to the crust. *Lithos* **198-199**, 117–128 (2014).

84. J. A. Crisp, Rates of magma emplacement and volcanic output. *J. Volcanol. Geotherm. Res.* **20**, 177–211 (1984).
